# Genomic Epidemiology and Characterization of Methicillin-Resistant *Staphylococcus aureus* from Bloodstream Infections in China

**DOI:** 10.1128/mSystems.00837-21

**Published:** 2021-11-02

**Authors:** Ye Jin, Wangxiao Zhou, Qing Zhan, Beiwen Zheng, Yunbo Chen, Qixia Luo, Ping Shen, Yonghong Xiao

**Affiliations:** a State Key Laboratory for Diagnosis and Treatment of Infectious Diseases, National Clinical Research Center for Infectious Diseases, Collaborative Innovation Center for Diagnosis and Treatment of Infectious Diseases, The First Affiliated Hospital, Zhejiang University School of Medicine, Hangzhou, China; b Infection Control Department, The First Affiliated Hospital, Zhejiang University School of Medicine, Hangzhou, China; University of Illinois at Chicago

**Keywords:** MRSA, epidemiology, genomics, evolution, phylogenetic reconstruction

## Abstract

Since 2010, methicillin-resistant *Staphylococcus aureus* (MRSA) ST59 began to increase in prevalence in China, gradually replacing ST239 and has become the dominant clone in most hospitals in China. Here, we investigated the changing epidemiology, phylogenetic reconstruction, and genomic characterization of MRSA clones in China to identify the genomic driving factors in the prevalence of ST59. Most MRSA isolates were identified as ST59 (36.98%; 277/749), which increased from 25.09% in 2014 to 35.53% in 2019. The phylogenetic analysis of the 749 MRSA isolates showed a high level of diversity and the copresence of hospital-associated, community-associated, livestock-associated, and hypervirulent clones. Furthermore, minimum spanning trees revealed that ST59 MRSA clones from different hospitals and regions were integrated, suggesting that frequent exchanges had occurred between regions and hospitals. ST59 clones displayed higher susceptibility to antimicrobials than did ST239 and ST5 MRSA clones, indicating that resistance to non-β-lactam and fluoroquinolone antibiotics may be not critical for the epidemic success of ST59 clones. Virulence factors detection showed that *sak* and *chp* genes enriched in MRSA ST59 may be associated with the enhanced spreading success of ST59, whereas *qacA* may have contributed to the predominance of ST5 in East China. Our refined analysis of different clones among ST239, ST5, ST59, and ST398 demonstrated the existence of potential driving factors for the evolution of nosocomial MRSA populations and diversity of the evolutionary events surrounding clonal replacement.

**IMPORTANCE** As a developing country, China has an unbalanced health care system due to regional differences in economic development. However, China is also a country worthy of study with regard to the population dynamics of MRSA within the more resource-rich health care systems. In this study, we carried out genomic analysis to investigate the genomic epidemiology and characterization of MRSA isolated from bloodstream infections over a timespan of 6 years. Our refined analysis of different MRSA clones among ST59, ST5, ST239, and ST398 demonstrated the existence of driving factors for the evolution of nosocomial MRSA populations and diversity of the evolutionary events surrounding clonal replacement.

## INTRODUCTION

Staphylococcus aureus is a commensal bacterial species that can colonize the skin and mucous membranes of the human body. It can cause a multitude of infections, ranging from mild skin lesions to more dramatic forms of systemic infections (1). Since it was first identified in the United Kingdom in the 1960s, methicillin-resistant S. aureus (MRSA) has become one of the most successful pathogens in most health care settings and hospitals worldwide (2). Infections caused by MRSA are generally associated with higher mortality than infections caused by methicillin-susceptible S. aureus. In addition, MRSA strains can lead to an increase in hospitalization costs and the extension of the length of hospital stays (3, 4). Moreover, since they were first described in 1980s, community-acquired MRSA (CA-MRSA) infections have been rising in frequency (5, 6). CA-MRSA was once traditionally limited to healthy people outside the hospital setting, which was the basis for differentiating CA-MRSA from HA-MRSA. However, in the last decade, a blurring of the definition between CA-MRSA and HA-MRSA has been observed. CA-MRSA has gradually gained a foothold in health care settings, and the more susceptible CA-MRSA clones have gradually replaced the multiresistant HA-MRSA lineages in several countries, including China (7–11). This indicates that the epidemiological dynamics of MRSA is associated with the increasing transmission of CA-MRSA strains in health care settings.

In 2005, the percentage of MRSA strains isolated from inpatients in China increased to 69.0%. However, with the Chinese government’s introduction of stringent infection control legislation, the isolated rate of MRSA has also declined annually and has rapidly decreased to 31.4% in China in 2019 (12). A previous study from Shanghai, China, indicated that the prevalence of ST239 clones had declined more sharply than that of other clones (13), indicating that some unknown factors or molecular processes responsible for this decline of ST239 clone exist.

Although infections caused by MRSA occur globally, there is no unifying or single pandemic clone prevalent all over the world. MRSA strains have been able to evolve into various lineages (14), and with the emergence of new adapted clones, previously successful and wildly disseminated clones started to decline and become less dominant and were finally replaced by the former adapted clones (15, 16). For instance, the predominant HA-MRSA clone EMRSA-16 (CC30) in the United Kingdom was replaced by EMRSA-15 (CC22) in the early 2000s (16). Likewise, USA400 (ST1), once the most dominant CA-MRSA lineage in the United States, lost its dominance at the turn of the century, and then USA300 (ST8) replaced it, becoming the most prevalent clone in the United States (17).

Due to the constantly changing epidemiology of MRSA, monitoring the characteristics of MRSA strains and the transmission routes of emerging clones is required. Hence, to better understand the changing epidemiology of MRSA clones for the purpose of guiding new control initiatives, we conducted a laboratory-based multiregion surveillance study. A total of 61 hospitals across 37 cities in 18 provinces were involved in the present study, and a total of 749 MRSA isolates were collected from patients with bloodstream infections during the 6-year study period from 2014 to 2019. Here, we characterized the epidemiological and genomic investigation of MRSA bloodstream infections in China. Whole-genome sequencing (WGS) can help to elucidate the reasons for outbreaks and identify emerging MRSA lineages by permitting better distinguishing capacity (18, 19). We reconstructed the phylogenies of the most frequently sampled lineages in this study (ST59, ST5, ST239, and ST398) and investigated variations in the clinical molecular characteristics of these clones. We further described the increased prevalence of ST59 clones in bloodstream infections. Overall, our results demonstrate the complex epidemiological dynamics and benefits of genome-informed surveillance of MRSA strains in China.

## RESULTS

**FIG 1:**
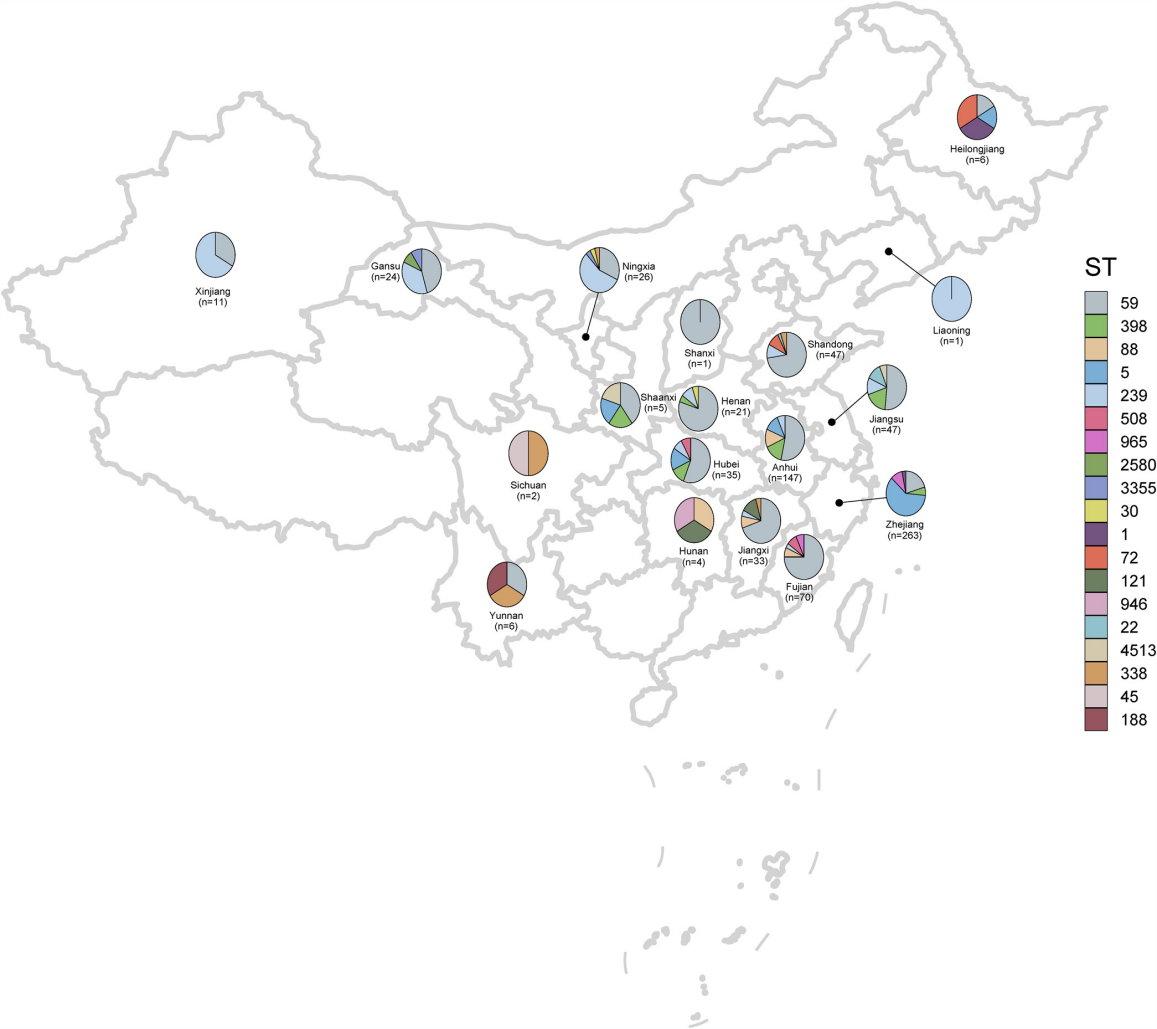
Clonal distribution of the 749 MRSA isolates in 61 hospitals from 2014 to 2019 included in this study. The map was created by using the corresponding map data with R package ggplot2 (https://github.com/tidyverse/ggplot2). The world map data were directly obtained from the R package maps v3.3.0 (https://github.com/adeckmyn/maps), which was imported from the Natural Earth data project (the 1:50m world map, version 2.0, the latest version available in 2015).

China has 34 provincial administrative regions, with a population of over 1.4 billion. In the present study, we focused on the epidemiology, evolutionary dynamics, and whole-genome genetics of 749 MRSA strains collected between 2014 and 2019 from 61 hospitals across the six administrative regions of China (Fig. 1). The strains isolated in each region were distributed as follows: east, 15 hospitals and 427 MRSA strains; middle, 21 hospitals and 240 MRSA strains; northwest, 4 hospitals and 35 MRSA strains; north, 4 hospitals and 32 MRSA strains; northeast, 2 hospitals and 7 MRSA strains; and southwest, 2 hospitals and 8 MRSA strains. All MRSA isolates were evaluated for antibiotic susceptibility and were subjected to WGS analysis.

### Genome sequencing highlighted the population structure of MRSA isolated from bloodstream infections in China from 2014 to 2019.

To study the accurate population structure of MRSA isolated from bloodstream infections from 2014 to 2019, we first performed a whole-genome phylogenetic analysis. In total, 35 STs were identified, and 34 isolates were defined as new singletons. As shown in Fig. 1, as estimated from our laboratory networks, the most common MRSA ST isolated from bloodstream infection identified was ST59, with prevalence rate of 36.98%, which varied from 17.86 to 80.95% across different administrative provinces. ST5 was the second most common ST in this study and was present at the highest frequency in Zhejiang Province in east China (56.36%, 133/265) and at frequencies of <5% in other provinces, except for Anhui Province (8.84%, 13/147). ST5 MRSA clones have been described as a representative newly emerging epidemic clone in east of China, and a high prevalence in Shanghai and Zhejiang Province has been reported (13, 20). ST239 accounted for 7.34%. This ST was more prevalent in north and northwest China, with prevalence rates of more than 40.0%.

**FIG 2:**
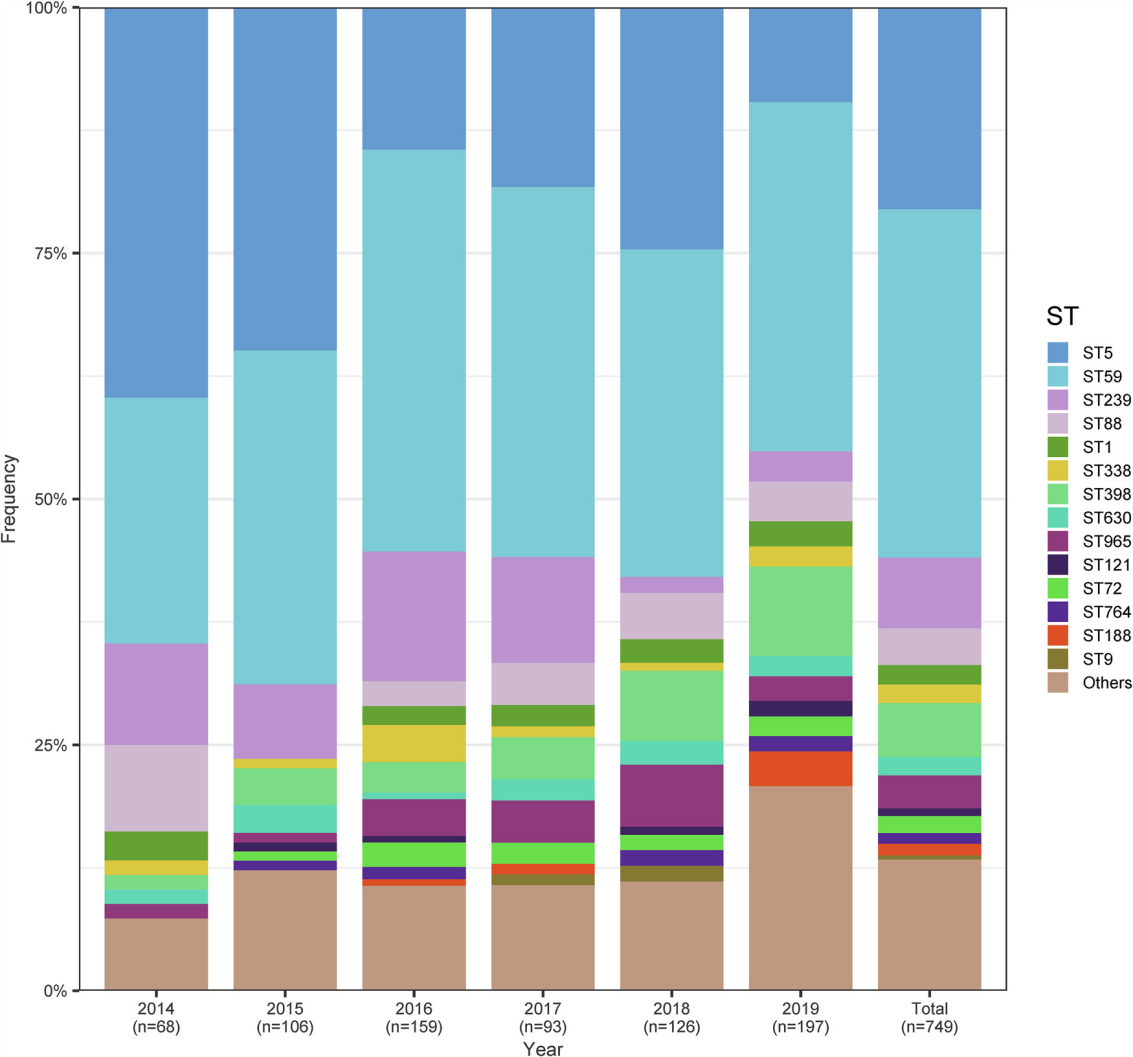
Sequence type distribution among MRSA isolates from 2014 to 2019.

It is important to emphasize that from 2014 to 2019, a higher strain-diversity occurred yearly for our MRSA isolates in the present study (see Fig. S1 in the supplemental material). As shown in Fig. 2, we found that the percentage of sporadically occurring MRSA isolates such as ST30, ST4513, ST1821, and ST5529 identified among the BSIs in China increased from 5.88% in 2014 to 20.30% in 2019. Notably, ST59-SCC*mec* IV clones continued to predominate in China, but the prevalence of sporadically occurring MRSA of different STs from BSIs increased 5-fold between 2014 and 2019. Furthermore, an increasing diversity of staphylococcal chromosomal cassette *mec* (SCC*mec*) elements in the sporadic MRSA isolates was also detected, including seven different SCC*mec* types and subtypes (IVa, IVc, IVh, IVg, IVi, V, and VT). This finding indicated that sporadic MRSA isolates may constitute a significant potential reservoir for SCC*mec* elements that may, in particular, represent an extensive reservoir of virulence and resistance genes located on mobile genetic elements (MGEs).

10.1128/mSystems.00837-21.1 FIG S1 Diversity of 749 MRSA from 2014 to 2019. Download FIG S1, PDF file, 0.02 MB. Copyright © 2021 Jin et al. 2021 Jin et al. https://creativecommons.org/licenses/by/4.0/ This content is distributed under the terms of the Creative Commons Attribution 4.0 International license.

**FIG 3:**
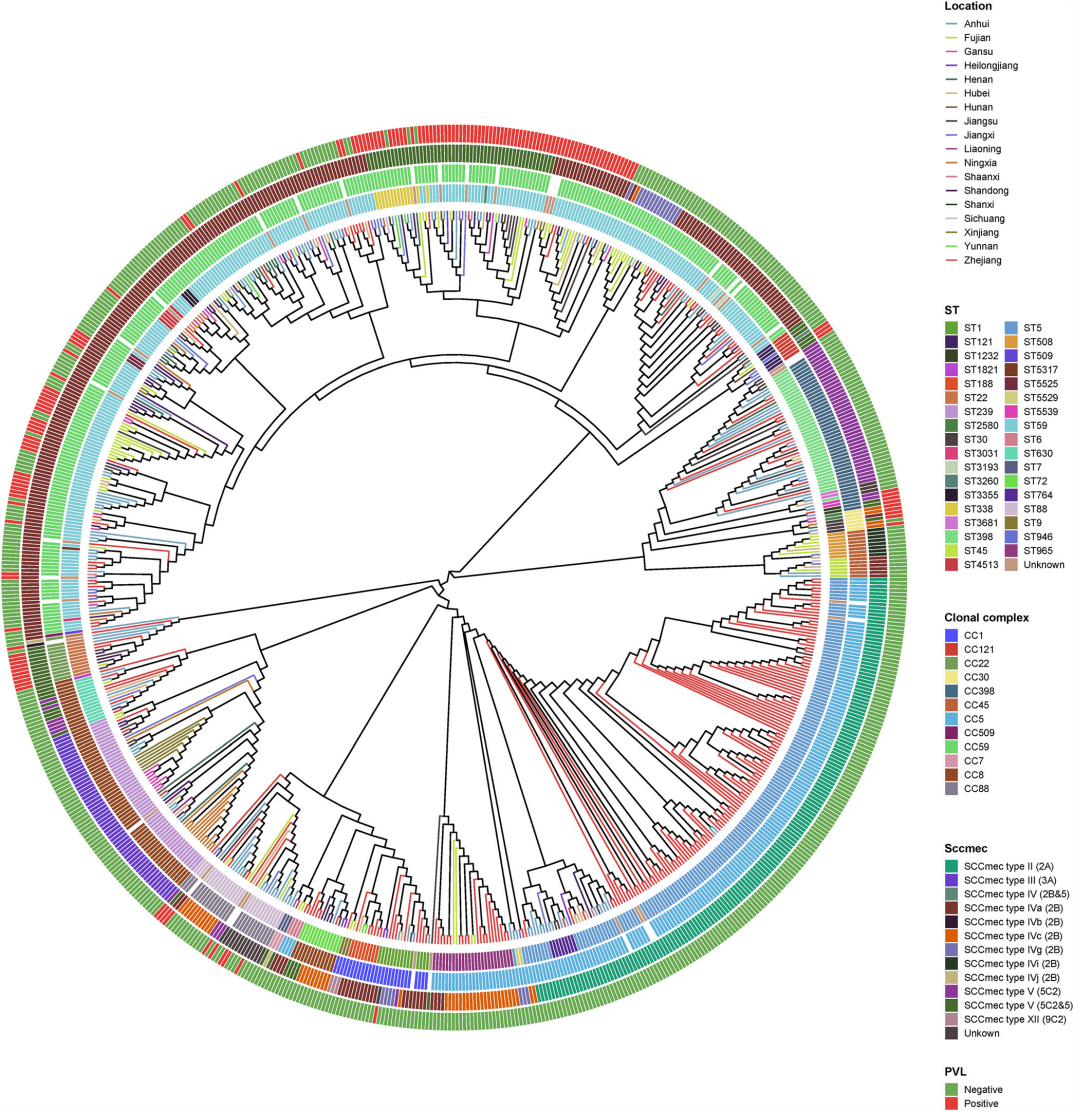
Phylogenetic tree of all 749 MRSA. Locations are distinguished by different colors with lines. ST types, clonal complex types, SCC*mec* type, and PVL presence are color-coded in the inner rings.

We observed that, despite this diversity, the prevalence of STs changed significantly among the MRSA strains from 2014 to 2019. The proportion of ST59 MRSA clones increased from 25.09% in 2014 to 35.53% in 2019. Interestingly, a slight but significant increased pattern was also found for ST398 clones (from 1.47% in 2014 to 9.14% in 2019). Notably, the proportion of ST5 MRSA clones exhibited a dramatic decline from 39.71% in 2014 to 9.64% in 2019. A similar descending trend was also found in ST239 MRSA clones, which dropped remarkably from a 10.29% prevalence rate in 2014 to 3.05% in 2019. The 34 identified ST types belong to 16 clonal complexes (CCs), with the four epidemic CCs (CC59, CC5, CC8, and CC398) comprising more than 78% of all 749 MRSA strains (Fig. 3).

In addition, five SCC*mec* types (types II to V and type XII) were identified in all MRSA strains, and an additional 17 isolates were nontypeable. In this study, SCC*mec* type IV predominated in all administrative provinces and accounted for 48.73% (365/749) of 749 MRSA strains. This cassette is often associated with ST59 and its variant such as ST338 and ST965. SCC*mec* IV has been widely transmitted in the last 10 years in China, gradually replacing the previously more epidemic hospital-acquired SCC*mec* types II and III (21, 22). Another SCC*mec* type that has been transmitted in recent years in China with a similar pattern was SCC*mec* V, the third most epidemic cassette type in our study (*n* = 145, 19.46%) next to the more traditionally hospital-associated SCC*mec* III (*n* = 55, 7.34%) (23, 24). Almost all SCC*mec* V are associated with CC59, C398, CC22, and CC121. Type II cassettes were also abundant (*n* = 164); notably, all of them belonged to CC5 and were particularly prevalent in the Zhejiang Province (rates of 21.90%) but were rare in other regions (0.21 to 8.03%). In addition, three isolates carried the SCC*mec* type XII, and all of them belonged to ST9.

### Copresence of hospital-associated, community-associated, livestock-associated, and hypervirulent clones.

We then combined the three characterization methods (multilocus sequence typing [MLST], SCC*mec* typing, and *spa* typing) to investigate specific lineages with specific typing combinations (see Table S1). The most prevalent were the Asian-Pacific clone ST59-SCC*mec* IV (*n* = 231, 30.84%) and the New York/Japan ST5-SCC*mec* II clone (*n* = 154, 20.56%), followed by the ST239-SCC*mec* III (*n* = 54, 7.21%) clone and the Panton-Valentine leucocidin (PVL)-positive Taiwan clone ST59-SCC*mec* V (*n* = 34, 4.54%). Based on detailed clinical information, 10 ST59 clones from Zhejiang Province and 13 ST338 clones (25) were confirmed as CA-MRSA, while 73 ST5 clones and 2 ST239 clones from Zhejiang Province were confirmed to be HA-MRSA isolates. However, due to a lack of available clinical records, other strains could not be identified as CA-MRSA or HA-MRSA. In this study, the most common lineage was ST59. In fact, the most predominant CA-MRSA lineage in Asia was ST59 (26). Indeed, there is mounting evidence that the increasingly more prevalent CA-MRSA clones were involved in health care-associated infections (27, 28). The growing numbers of ST59 clones emerging in Chinese hospitals identified in this study highlight the multi-aspect adaptive evolution of MRSA as a pathogen, which is no longer limited to health care environments. Furthermore, mathematical models have previously suggested that CA-MRSA will eventually replace the traditional HA-MRSA strains in health care settings (27, 29).

10.1128/mSystems.00837-21.4 TABLE S1 Metadata of 749 MRSA from 2014 to 2019. Download Table S1, DOCX file, 0.2 MB. Copyright © 2021 Jin et al. 2021 Jin et al. https://creativecommons.org/licenses/by/4.0/ This content is distributed under the terms of the Creative Commons Attribution 4.0 International license.

Moreover, several other clones, including the globally disseminated hypervirulent clone ST121-SCC*mec* V PVL+ (*n* = 3), have also been identified. As a virulent clone, ST121-MRSA has spread across Africa, Asia, and Europe, with an overall prevalence rate of <5% around the world. In addition, we determined that 41 isolates (*n* = 34, 4.54%) were identified as livestock-associated MRSA (LA-MRSA) ST398 clones. Although, in the present study, the prevalence of ST398-MRSA was still low, it showed an increasing trend. Moreover, several studies have reported cases of CA or HA infections caused by ST398 without livestock contact (30–33), suggesting that the LA ST398 clone is gaining access to community and health care environments.

Another LA-MRSA clones, ST9 (*n* = 3) were also found in this study, which is the major cause of bovine mastitis in Asia (34, 35). However, although mild or severe infections caused by ST9 have been reported in China (36–38), there is still very limited information on the occurrence of infections by ST9 clones in humans. Taken together, the growing morbidity of LA-MRSA isolates in human infections emphasizes the existence of reservoirs of LA-MRSA clones that could become epidemic in both community and nosocomial settings might have been underestimated.

### Phylogenetic reconstruction of the four major lineages.

**FIG 4:**
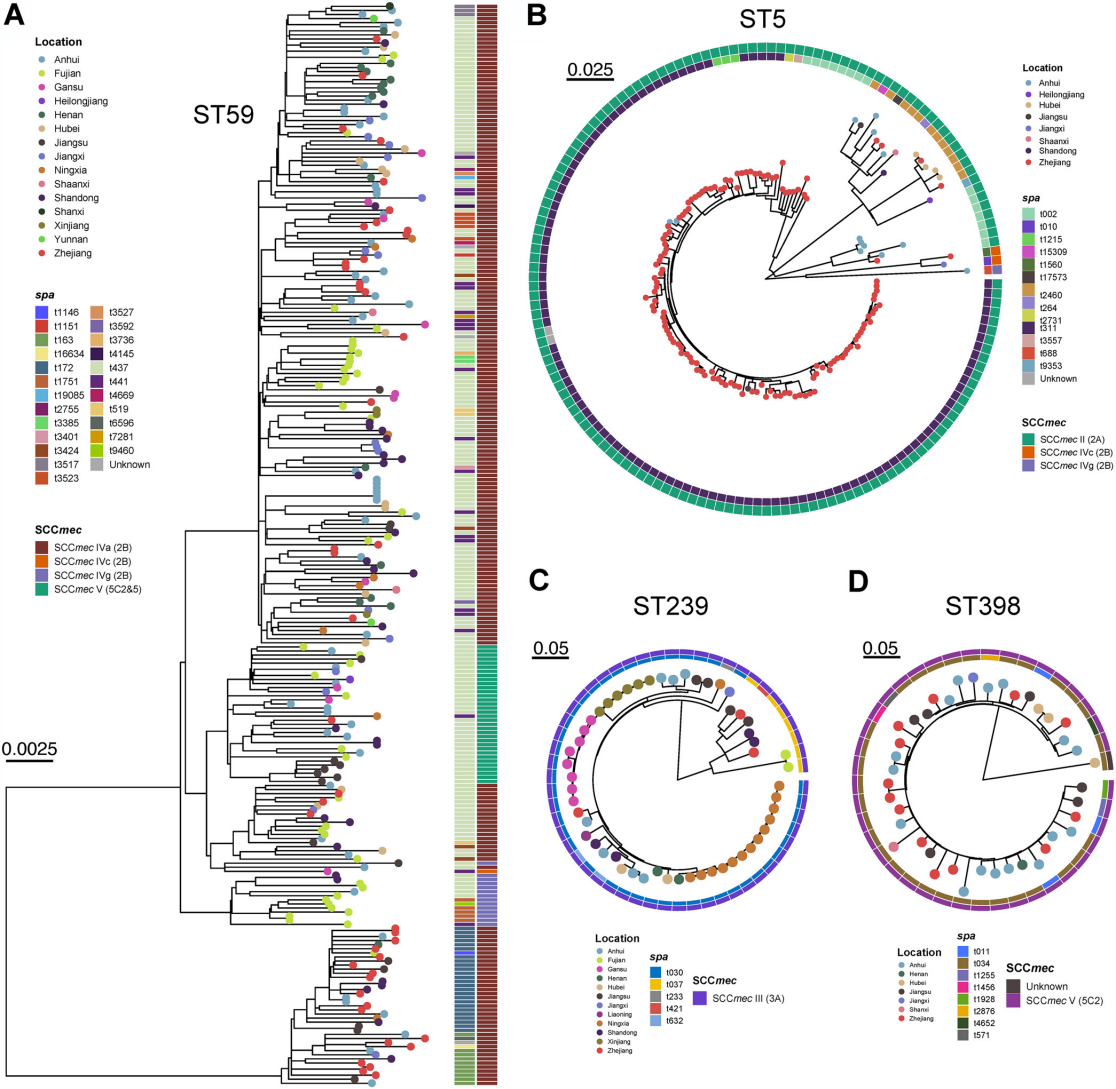
Phylogenetic reconstruction of the major four lineages (ST59, ST5, ST239, and ST398) in this study. The different locations are distinguished by circle color. The SCC*mec* type and *spa* type of MRSA strains is shown on the left.

**FIG 5:**
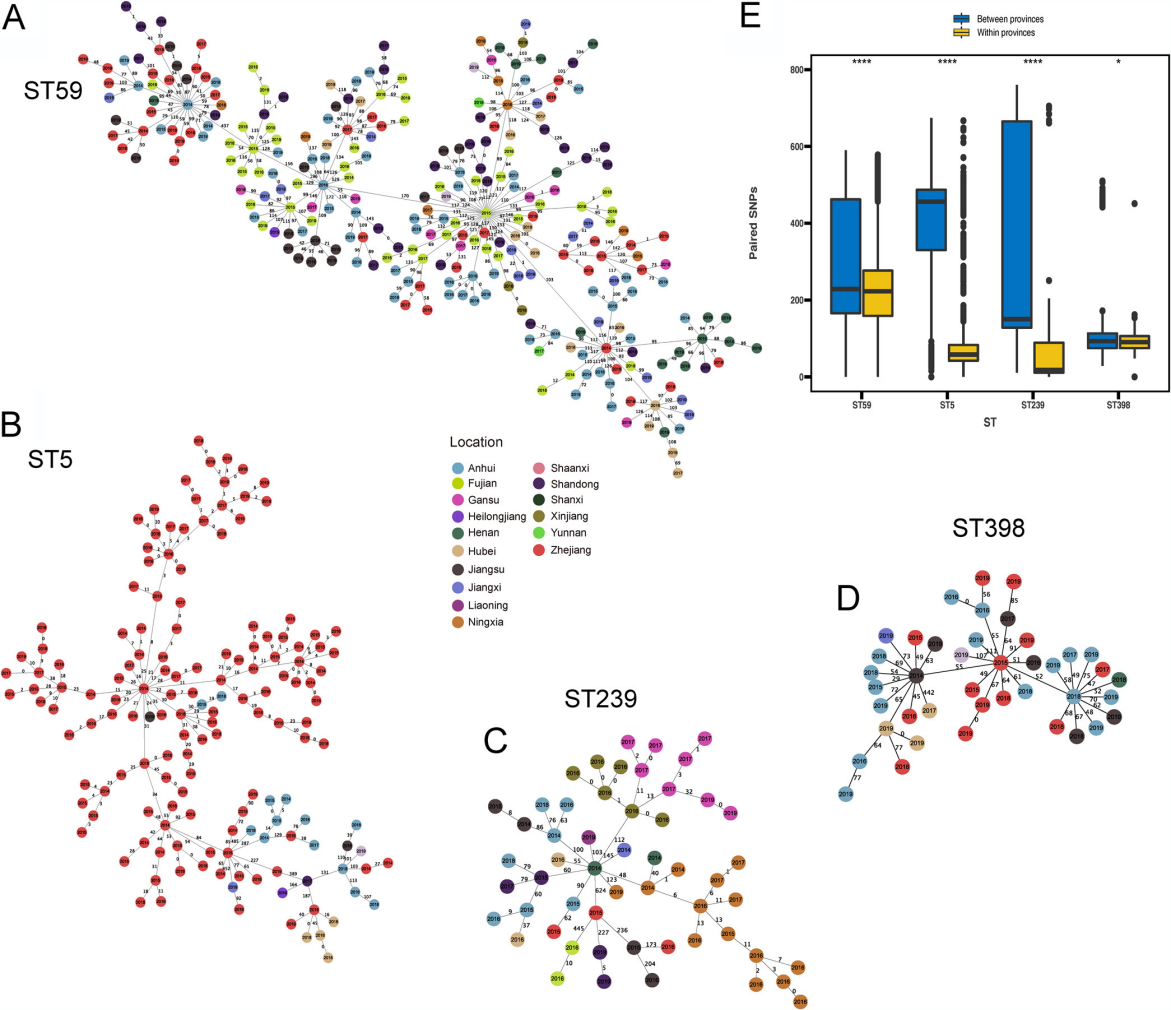
SNP distribution of the major STs. (A to D) MSTs of ST59, ST5, ST239, and ST398 MRSA strains based on core-SNP with MLST grouping. The different locations are distinguished by circle color. The SNP differences of each STs were shown on the connecting lines. (E) Paired SNPs among ST59, ST239, ST5, and ST398 MRSA isolates. *, *P* < 0.05; ****, *P* < 0.0001.

We first constructed the phylogenetic trees of the major STs to investigate the heterogeneity of each ST. We found that the ST59 was more likely to spread between provinces that were geographically close (Fig. 4A). Compared to the ST59 lineage, ST239 and ST5 clones showed a greater trend for spreading within the province. To compare differences in SNPs among the common ST types, we constructed a minimum spanning tree (MST) based on core single nucleotide polymorphisms (SNPs) with MLST, which showed the relatedness varied among the clusters of different STs (Fig. 5). There were 0 to 590 SNPs among the 265 ST59 MRSA clones, with an average distance of 274 SNPs (Fig. 5A). In Zhejiang Province, the SNP differences for ST59 ranged from 0 to 578 (median = 260, mean = 329). For example, in Anhui Provinces, the SNP differences of ST59 ranged from 0 to 570 (median = 226, mean = 259). Moreover, the SNP differences between Anhui Province and Zhejiang Province ranged from 29 to 584 (median = 242, mean = 321). From a previous study, <24 SNP differences or >40 SNPs could be applied to define isolates as being likely or unlikely to be involved in putative recent transmission events (29). Therefore, transmission within and between the provinces for most ST59 MRSA clones is not occurring in tightly linked clusters, but it is likely that the ST59 MRSA clones have circulated in the community for a long time. As shown in Fig. 5A, in the ST59 MST, ST59 MRSA clones from different hospitals and regions overlapped, suggesting that frequent exchanges had occurred between regions and hospitals. However, fewer SNP differences were found among the 154 ST5 MRSA clones, with an average distance of 70 alleles, compared to the ST59 MRSA clones. Remarkably, ST5 was observed to have the greatest frequency in the Zhejiang Province in this study, and most ST5 clones were closely related (<40 SNPs) (Fig. 5B). As shown in Fig. 5C, there were 0 to 760 SNPs within the 54 ST239 clones (median = 147, mean = 267). Notably, in Xinjiang, Gansu, and Ningxia Provinces, most ST239 clones differed from each other by <20 SNPs. Furthermore, MST analysis showed ST239 clones from the Xinjiang Provinces were more closely related to the MRSA ST239 clones from Gansu Province (<24 SNPs) than the ST239 clones from other provinces. Interestingly, Xinjiang and Gansu Provinces were geographically close, suggesting that clonal transmission of ST239 clones had occurred between the two provinces. For the 41 ST398 clones identified, there were approximately 0 to 510 SNP differences (median = 92, mean = 111), with an average distance of 274 SNPs. In all, widespread transmission based on these SNP patterns is likely for ST59 but less likely for ST5 and ST239 clones, which matches the putative wider environments in which these clones spread, e.g., in the community for ST59 versus in hospitals for ST5 and ST239 clones.

### MRSA ST59 exhibited more susceptibility to antibiotics than did MRSA ST239 or ST5.

**FIG 6:**
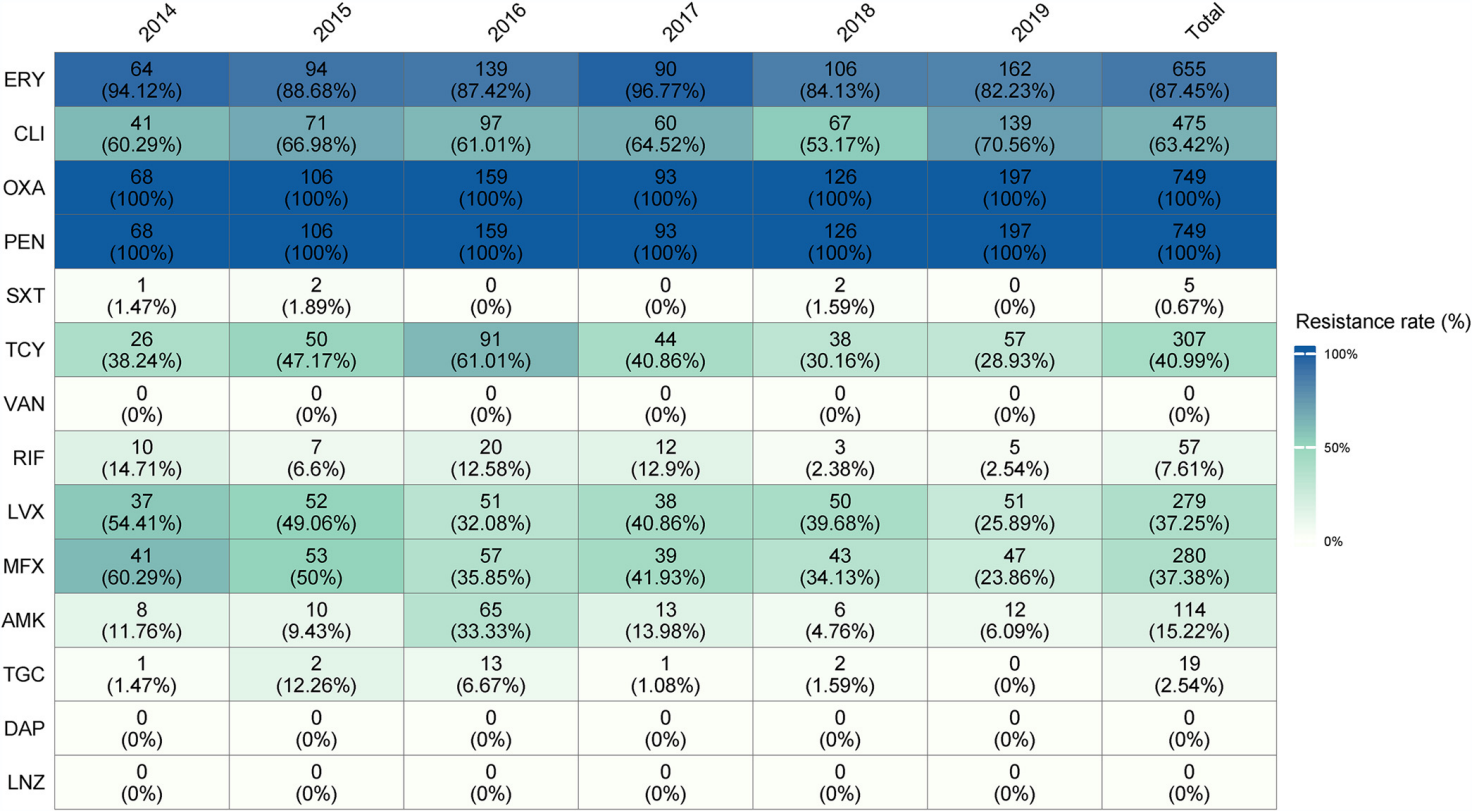
Antimicrobial resistance profiles among 749 MRSA isolates from 2014 to 2019.

**FIG 7:**
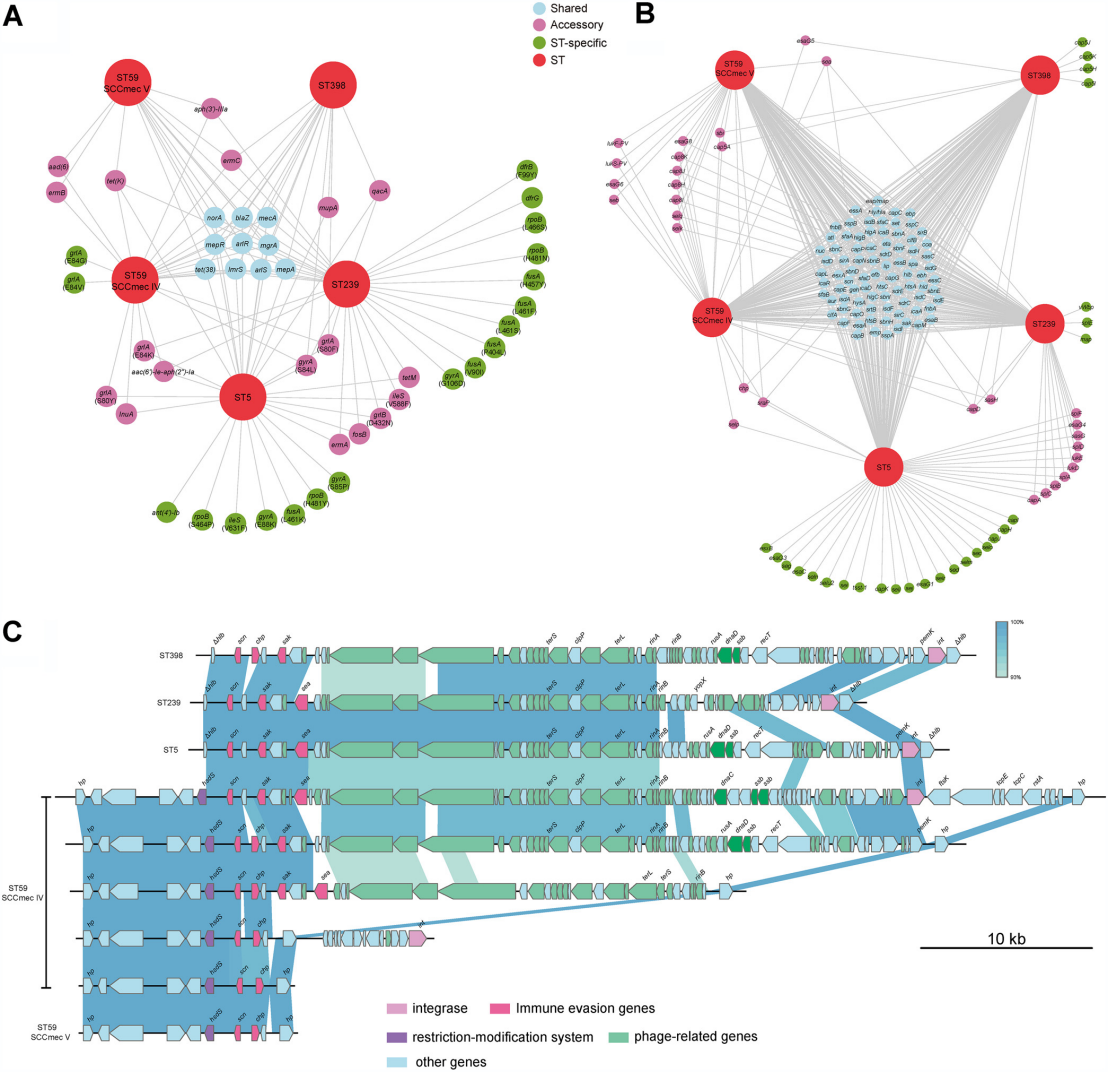
Distribution of drug resistance profiles (A) and virulence factors (B) of ST59 isolates in comparison to MRSA ST239 and ST5 clones. (C) Comparison of the vSaβ among ST59, ST5, and ST239 isolates. Arrows and arrowheads represent open reading frames (ORFs) and the direction of transcription, respectively.

S. aureus can easily acquire various resistance genes. Given the extreme importance of resistance to antimicrobials in MRSA, we next evaluated antimicrobial resistance and the presence or absence of resistance genes (Fig. 6 and 7A; see also Fig. S2).

10.1128/mSystems.00837-21.2 FIG S2 Distribution of mutations, antimicrobial-resistant genes, and MGEs in major STs. Download FIG S2, PDF file, 1.1 MB. Copyright © 2021 Jin et al. 2021 Jin et al. https://creativecommons.org/licenses/by/4.0/ This content is distributed under the terms of the Creative Commons Attribution 4.0 International license.

**FIG 8:**
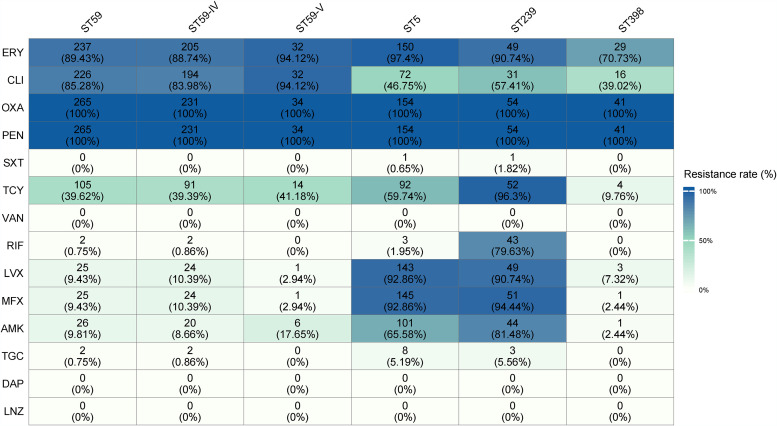
Antimicrobial resistance profiles among different STs (ST59, ST5, ST239, and ST398).

In the present study, no resistance to linezolid, vancomycin, or daptomycin was detected for any of the isolates, which correlated with the predicted genotypes. As expected for the aforementioned antimicrobial agents, trimethoprim-sulfamethoxazole exhibited the best antimicrobial activity (99.33% sensitivity in MRSA), while erythromycin displayed the poorest antibacterial activity (12.55% sensitivity in MRSA). As expected, most of ST59, ST5, ST239, and ST398 clones in this study carried *erm* genes showing resistance to erythromycin. Moreover, erythromycin resistance genes *ermA*, *ermB*, and *ermC* displayed different pattens in different ST types of MRSA: *ermA* was detected only in ST5 clones, while *ermB* was detected only in ST59 clones. Interestingly, none of the ST5 MRSA clones carried *ermC* (Fig. 5A; see also Fig. S2). There was a strong relatedness between molecular typing and antimicrobial resistance profiles (Fig. 8). Resistance to moxifloxacin, rifampin, and levofloxacin by chromosomal mutation was specific to a particular MRSA lineage. Specifically, fluoroquinolones showed poor activity against MRSA ST239 and MRSA ST5 (resistance rate >90.0%), while ST239 and ST5 clones had higher rates of resistance to fluoroquinolones (moxifloxacin and levofloxacin than MRSA ST59 clones (<11.0%). Notably, we found that mutations in *grlA* and *gyrA* genes could lead to fluoroquinolone resistance, i.e., we observed that the quinolone resistance *grlA* S80F mutation was present in most quinolone-resistant ST59 and ST239 clones, whereas *grlA* S80Y and E84K mutations were present in most ST5 clones, suggesting that such nucleotide mutations in the *grlA* gene may also impact fitness of MRSA ST59 clone. Further, the rates of resistance to rifampin of MRSA ST239 were much higher than all other ST types, consistent with the results of the predicted genotypes: the rifampin resistance *rpoB* H481N and *ropB* L466S mutations were only detected in a majority of rifampin-resistant ST239 clones.

Also, resistance to some antibiotics through horizontal gene transfer was specific to a particular MRSA lineage. For instance, the rates of resistance to tetracycline of ST239 clones were much higher than all other ST types, since a low proportion of ST59 and ST5 MRSA clones harbored *tetK*, while *tetM* was only detected in the few ST5 clones and a majority of ST239 clones. The resistance rates of MRSA ST239 and ST5 clones to amikacin were significantly higher than those for ST5 and ST398, which could be explained by the high carriage rate of *aac(6′)-Ie-aph(2″)-Ia* in the former clones. In addition, the lincosamide resistance gene *lnuA* and the fosfomycin resistance gene *fosB* were detected in most ST5 MRSA clones, and most *qacA*-positive MRSA strains belonged to the ST5-SCC*mec* II clones (94.06%, 95/105).

### Different patterns of virulence genes among ST59 and other major STs types.

The PVL genes *lukS* and *lukF* were detected in 148 MRSA isolates (19.76%) (Fig. 7B; see also Fig. S3). The majority (63.51%) of PVL-positive isolates were identified as ST59 (94 of 148 isolates), including in 97.06% (33 of 34 isolates) of ST59-SCC*mec* V and in 23.02% (61 of 265 ST59 clones) of ST59-SCC*mec* IV. Furthermore, emerging MRSA isolates such as ST22 and ST338 all belonged to PVL-positive isolates. No PVL-positive isolate belonged to ST239, ST5, and ST398 clones.

10.1128/mSystems.00837-21.3 FIG S3 Distribution of virulence genes in major STs. Download FIG S3, PDF file, 2.2 MB. Copyright © 2021 Jin et al. 2021 Jin et al. https://creativecommons.org/licenses/by/4.0/ This content is distributed under the terms of the Creative Commons Attribution 4.0 International license.

The distribution of *sak* showed significantly different patterns across different STs. In the present study, almost all ST59-SCC*mec* IV clones carried *sak*, *scn*, and *chp* genes. This indicated that most ST59-SCC*mec* IV clones belonged to IEC (immune evasion cluster) type B. The S. aureus IEC is encoded by β-hemolysin-converting bacteriophages (βC-Φs), which has seven variants (IEC types A to G) (39). All IEC variants carry *scn* and a different combination of *sea*, *sak*, and *chp*. These genes encode the human-specific immune modulators staphylococcal enterotoxin A (SEA), staphylokinase (SAK), and chemotaxis inhibitory protein of S. aureus (CHIPS) (40). However, almost all the ST59-SCC*mec* V clones (33/34) and all ST398 clones lacked the *sak* gene, indicating that ST59-SCC*mec* V and ST398 clones belonged to IEC type C.

ST239, ST5, and ST59-SCC*mec* carried the SAK-encoding prophage ΦSa3 that usually integrates into the *hlb* gene, whereas in ST59-SCC*mec* V and ST398 clones, the prophage was inserted into the genomic island νSaβ (Fig. 7C). However, compared to ST59 and ST398, regardless of ΦSa3 or νSaβ, most ST5 clones (107/154, 124/154) harbored the associated enterotoxin A gene (*sea*), *scn*, and *sak* genes. However, nearly all ST5 clones (147/154) lacked the *chp* gene. Notably, the majority of ST239 clones (50/54) carried the *sea*, *sak*, and *scn* genes, but none of the ST239 clones carried the *chp* gene. In addition, enterotoxin genes such as *sec*, *seg*, *sei*, *sell*, *selm*, *seln*, *selo*, and *selu2* and the superantigens *tsst-1* were ubiquitous in ST5.

## DISCUSSION

Since China is a developing country with vast territories, the economic development of each region of China varies greatly due to regional differences. There is also a large gap in medical facilities and health care system between the eastern coastal areas and the northwestern regions. However, China is also a country worthy of study with regard to the population dynamics of MRSA within more resource-rich health care systems. We investigated the genomic epidemiology and characterization of MRSA isolated from bloodstream infections over a timespan of 6 years by WGS. According to the genomic analysis, we found a significant increase in ST59 MRSA clones that isolated from bloodstream infections, which have gradually been replacing ST239 and ST5 clones and have become the predominant clone in MRSA bloodstream infections.

Before 2000, in China, most MRSA strains isolated from hospitals were ST239-SCC*mec* III, which corresponded to the Hungarian pandemic lineage (41, 42). However, since about 2010, the ST59 clone began to increase in prevalence, gradually replacing ST239 and becoming the dominant clone in most hospitals in China (21, 22). To date, the success of ST59 in health care settings is still poorly understood. Thus, we further investigated the genomics and population structure of MRSA during the period of lineage replacement in China to look for evidence of the evolution of major lineages. We found that ST59 MRSA clones from different hospitals and regions, compared to the four phylogenies, were integrated, suggesting that frequent exchanges had occurred between regions and hospitals. Furthermore, ST59 also displayed a greater overall phylogenetic diversity than did other STs clones, and of note, compared to other STs, most ST59 MRSA clones exhibited higher adaptability within the hospitals, suggesting that the ST59 lineage underwent multiple and continuous evolutionary events. ST239-SCC*mec* III and ST5-SCC*mec* II once were the predominant HA-MRSA lineages in China (23, 24, 42). Compared to types II and III, MRSA-IV and MRSA-V strains harbored smaller SCC*mec* elements (24). Previous study has demonstrated that smaller SCC*mec* cassettes may reduce the fitness burden (25). Therefore, the increase in ST59 MRSA-IV and MRSA-V clones may contribute to harboring a smaller SCC*mec* cassette, which lead to lower potential fitness burden. Notably, fluoroquinolones are readily excreted in the perspiration through the pores of skin and can suppress the growth of bacteria, which inhibits S. aureus colonization in the skin and nose (43–45). However, the molecular characterization in this study and previous studies (13, 21) revealed that ST239 and ST5 clones, two previously pandemic lineages across China, were waning. There are several possible explanations for the success of a pandemic clone. One mechanism is antibiotic resistance and is the focus of national antibacterial management strategies. ST59 clones were more antimicrobial susceptible than were ST239 and ST5 MRSA clones, indicating that the resistance to non-β-lactam and nonfluoroquinolone antibiotics might not be critical for the successful epidemic spread of ST59 clones. Of note, there were lower numbers of antimicrobial resistance genes in ST59 than in ST239 or ST5 MRSA isolates. Thus, a critical factor in the success of epidemics of ST59 clones may be related to the innate physiological properties of this clone rather than to its resistance to antibiotics. Coculture experiments by Wang et al. showed that *in vitro* ST59 displayed higher growth rates and higher competitive forces than MRSA ST239 (21). In addition, Yu et al. also reported that ST59 isolate clones had a higher virulence capacity than did ST5 and ST239 clones (22). Thus, we suspect that ST59 clones may exhibit a better capacity for surviving outside the host compared to the replaced clones ST239 and ST5 and so promote ST59 transmission in hospitals.

In addition to ST59, compared to other regions in China, the ST5-SCC*mec* II clone is the most epidemic lineage in east China. However, the genetic basis for the successful dissemination of ST5 in east China is also still unknown. *qacA* encodes multidrug efflux pumps, which is associated with the resistance to or tolerance of chlorhexidine in S. aureus, and the increasing use of chlorhexidine for MRSA decolonization in hospitals has focused much attention on resistance to these antiseptics. HA-MRSA spreads primarily by hand transmission (44). Changes in hand washing and hygiene have been implemented in the last few decades, which formerly involved washing by soaps to the use of alcohol-based hand sanitizers and chlorhexidine (46). Therefore, most ST5 clones that harbored *qacA* may have had a competitive advantage in hospital environments in economically developed coastal regions in east China such as Zhejiang and Shanghai, where greater importance is attached to hand washing and hand disinfection (47).

There are also significant differences in the toxin profile among different MRSA lineages, especially for MGEs. MGEs can encode several toxins such as toxic shock syndrome toxin, PVL, enterotoxin, and exfoliative toxins. Thus, to identify the existence of potential genomic factors associated with the successful transmission of ST59, we next investigated the distribution of virulence genes among different MRSA clones. As a genomic island in *S. aureus*, νSaβ is an ancient component of the staphylococcal genome located at the same position in different *S. aureus* clones. νSaβ typically harbors a restriction-modification system, a set of enterotoxins, serine proteases, and bicomponent toxin. However, in most ST59-SCC*mec* IV, ST5, and ST239 clones, these factors have been replaced by an intact ΦSa3. In ST59-SCC*mec* V and ST398 clones, in contrast, most ΦSa3 genes have disappeared, leaving only the *chp* and *scn* genes retained. With the emergence of CA-MRSA strains, these clones have become increasingly important in hospital infections. It is therefore reasonable to postulate that the lack of the *sak* gene may be the driving force behind the emergence of the CA-MRSA clone in medical and health environments. In conclusion, the atypical integration of ΦSa3 in MRSA clones may promote strains to adapt to hospital settings, and the subsequent loss of ΦSa3 helps to disseminate the evolutional CA-MRSA clones more widely.

However, our study presented some limitations that should be considered. First, the number of MRSA strains collected from different hospitals and years was not equal, especially from hospitals in northeast and southwest China. This limitation may have influenced the integrality of MRSA epidemiology from hospitals or local regions and temporal analyses of clonal composition and limit the weight of the genomic epidemiological analysis, since although the phylogenetic tree could be constructed to determine the relative positions of any two MRSA isolates with degree of certainty, any unsampled populations missing from the tree may lead to an incomplete interpretation. Therefore, our study may not reflect the situation across China. Second, All MRSA isolates were isolated from bloodstream infections, so the sampling is highly biased toward MRSA strains from bloodstream infections and therefore is very likely not representative of the MRSA isolated from various samples in general. In addition, we did not address the precise contributions of the *sak* and *chp* genes surrounding clonal replacement. Further analysis and investigations should be performed to clarify how these genes mentioned above contribute to the success of the spread of ST59.

In conclusion, the displacement of ST239 by ST59 has occurred over that last decade, and *sak* and *chp* genes that were enriched in MRSA ST59 may be associated with the enhanced spreading success of ST59. In addition, we described the presence of geographically rarer MRSA ST5 lineages, which were more prevalent in Zhejiang Province but were scarce in isolates from other regions and may be associated with the *qacA* gene. Moreover, the prevalence of newly emerging LA-MRSA clones such as ST398 increased from 2014 to 2019, indicating the dissemination of such clones occurred in both community and hospital settings. Our study highlights a large diversity of ST types, SCC*mec* cassettes, and *spa* types for these major MRSA lineages, and furthermore, sporadic clones also result in a great variety of different lineages. Moreover, our refined analysis of the different clones among ST239, ST5, ST59, and ST398 demonstrated the presence of further resistance and virulence and diversity of the evolutionary events surrounding clonal replacement. Our data support the recommendations to clinicians regarding a multifactorial relationship between antibiotic use and resistance evolution: strict antimicrobial stewardship guidelines are still required.

## MATERIALS AND METHODS

### Study design and MRSA isolates.

This was a laboratory-based multiregion retrospective study of MRSA. The study was approved by the research ethics board at the First Affiliated Hospital of Zhejiang University, School of Medicine. Informed consent was waived since this project was a retrospective study and all individual patients were anonymized. A total of 61 hospitals in 37 large cities located across 18 provinces of China volunteered to participate in this study. A total of 749 nonrepetitive MRSA isolates were collected from patients reported to have a blood culture positive for MRSA between 2014 and 2019. We used the MALDI-TOF/MS to confirm the identities of the MRSA isolates.

The antimicrobial susceptibilities of all MRSA isolates were determined using agar dilution screening according to the guidelines established by the Clinical and Laboratory Standards Institute. The tested antimicrobial agents included oxacillin, penicillin, erythromycin, rifampin, tetracycline, trimethoprim-sulfamethoxazole, levofloxacin, moxifloxacin, teicoplanin, tigecycline, amikacin, linezolid, daptomycin, and vancomycin.

### WGS and genomic analysis of MRSA isolates.

We used the Ezup Column Bacteria Genomic DNA purification kit (Sangon Biotech, Shanghai, China) to extract the genomic DNA of all 749 MRSA strains. The sequencing library was prepared by using a Nextera XT kit (Illumina, San Diego, CA). Sequencing of the genomes was performed on a HiSeq X 10-PE150 platform (Illumina) by Novogene Co. (Beijing, China). The quality control of raw sequenced reads was performed using FastQC v.0.11.5 (https://www.bioinformatics.babraham.ac.uk/projects/fastqc/). After adaptor trimming and quality filtering (Phred quality score ≥20) by fastp v0.20.1 (48), the processed paired-end reads were assembled using shovill v1.1.0 pipeline (https://github.com/tseemann/shovill; based on SPAdes v3.14.1 [49]) using the default settings. Multilocus sequence typing of MRSA was identified by interrogating seven housekeeping genes of S. aureus using MLST software (https://github.com/tseemann/mlst) against the S. aureus typing database (50). MRSA isolates were assigned to clonal complexes (CCs) on the basis of sharing six of the seven alleles with the founder sequence type using PHYLOViZ v2.0 with the geoBURST Full MST algorithm (51). SCC*mec* and *spa* typing of MRSA strains was performed using the web-based SCC*mec*Finder (52) and SpaFinder (53) with default settings, respectively. Genome annotation of all MRSA isolates was performed with DFAST-core v1.2.11 (54).

### Phylogenetic analysis of MRSA isolates.

Roary v3.13.0 (55) was used to perform pangenome analysis with core gene alignment on all 749 MRSA isolates; an SNP alignment was then extracted from the core-genome alignment using snp-sites v2.5.1 (56), and a phylogenetic tree was constructed with RAxML v8.2.11 (57) using the ASC_GTRGAMMA substitution model (1,000 bootstrap replications). In addition, Snippy v4.6.0 (https://github.com/tseemann/snippy) was used to perform reference-based mapping and identify SNPs for major STs ST59, ST5, ST239, and ST398 clones with default parameters; M013 (GenBank accession no. CP003166), Mu3 (GenBank accession no. AP009324), TW20 (GenBank accession no. FN433596), and S0385 (GenBank accession no. AM990992) chromosomes were set as the references for ST59, ST5, ST239, and ST398, respectively. The recombined regions within the core genome were detected using Gubbins v2.4.1 (58). The phylogenetic trees for ST59, ST5, ST239, and ST398 isolates were constructed using the core SNPs from the recombination-free core-genome alignment with RAxML v8.2.11 (57) using the ASC_GTRGAMMA substitution model (1,000 bootstrap replications). All phylogenetic trees were rooted at the midpoint and visualized by ggtree package in R (59). The MSTs based on core SNP data from all ST59, ST5, ST239, and ST398 clones were generated using PHYLOViZ v2.0 with the geoBURST Full MST algorithm (51).

### Toxin genes, resistance genes, chromosomal mutations, and MGEs in MRSA strains.

The presence of virulence factors and resistance genes was detected by using ABRicate v1.0.0 (https://github.com/tseemann/abricate) with the CARD (60) and VFDB (61) databases with 80% identity and 80% query coverage cutoffs, respectively. PointFinder v3.2 (62) was used to identify antibiotic resistances encoded by chromosomal mutations with 90% identity and 90% query coverage cutoffs. Prophages and genomic islands were predicted using PHASTER (63) and IslandViewer 4 (64), respectively. We also used ComplexHeatmap package in R (65) to draw heatmaps with toxin genes, drug resistance genes, chromosomal mutations, and MGEs in MRSA strains.

### Data availability.

The sequence data of 749 S. aureus isolates have been deposited in the Sequence Read Archive database under BioProject accession no. PRJNA749878.
