# Supplementary figures and images for: Genomic Epidemiology and Characterization of Methicillin-Resistant Staphylococcus aureus from Bloodstream Infections in China
Source: mSystems. 2021 Nov 2;6(6):e00837-21. doi: 10.1128/mSystems.00837-21 (PMC8562482; doi:10.1128/mSystems.00837-21)

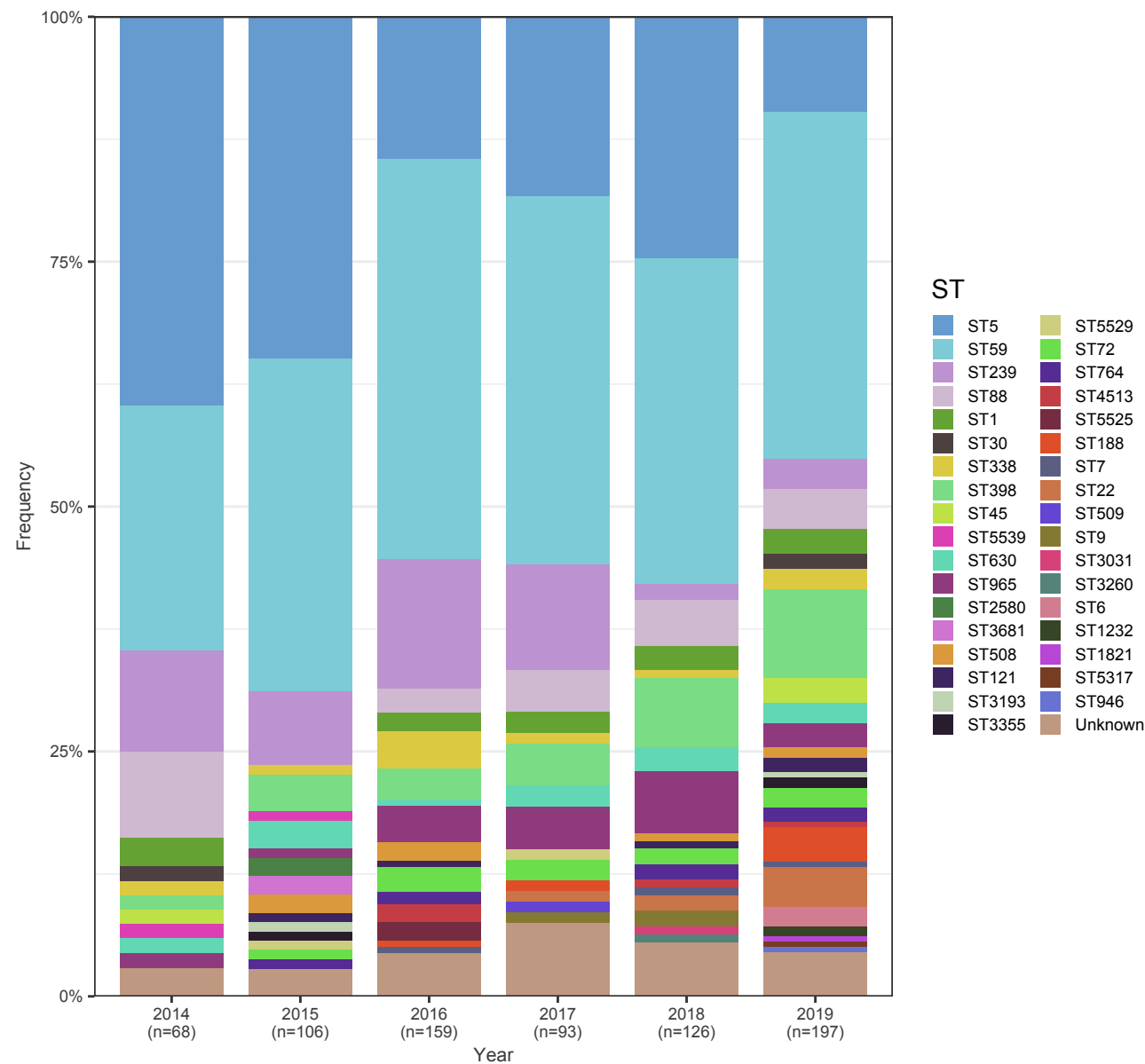

Supplement: FIG S1 [file msystems.00837-21-sf001.pdf]

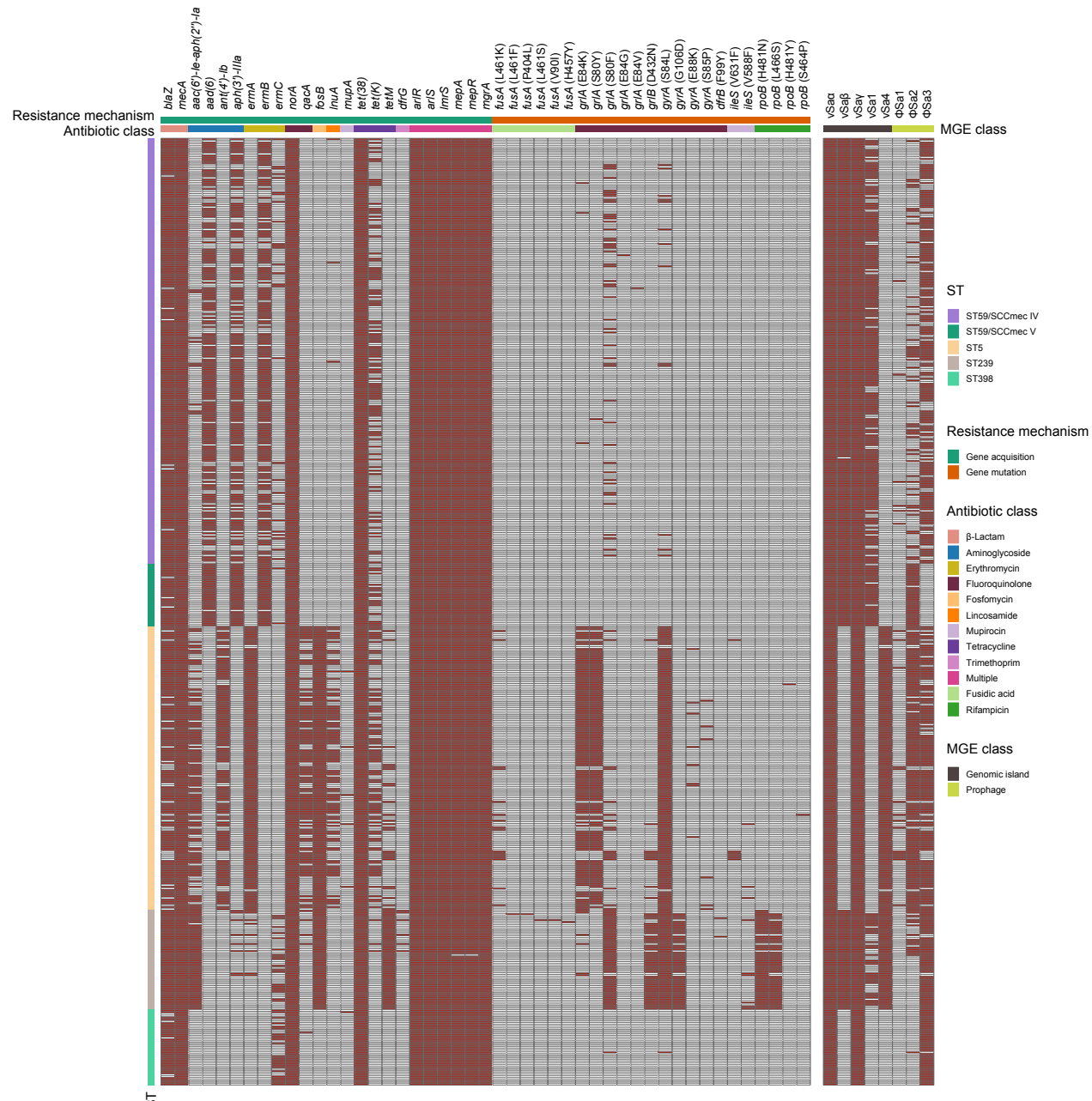

Supplement: FIG S2 [file msystems.00837-21-sf002.pdf]

ST

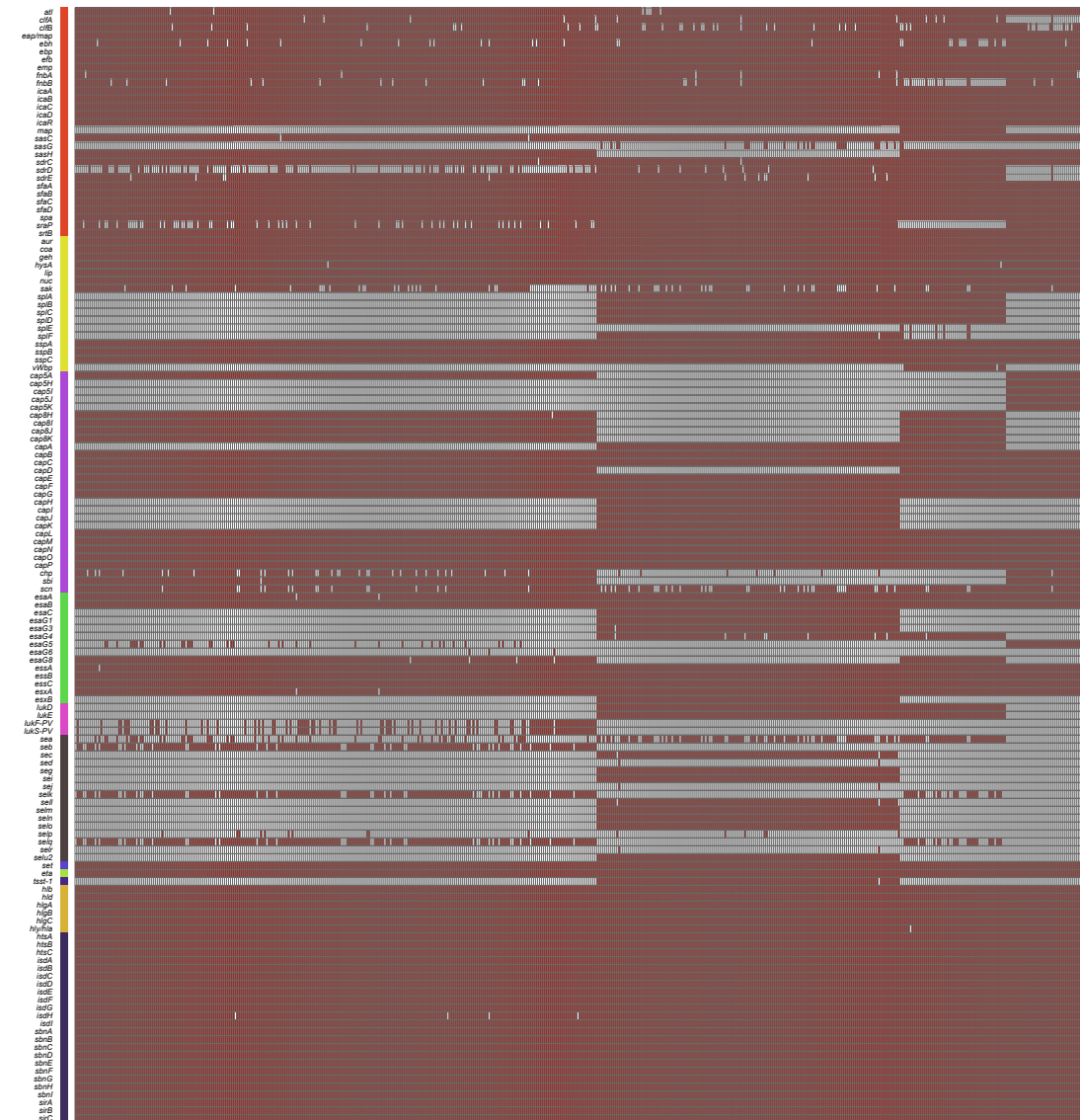

Supplement: FIG S3 [file msystems.00837-21-sf003.pdf]
