# Supplementary material for: Genomic Epidemiology and Characterization of Methicillin-Resistant Staphylococcus aureus from Bloodstream Infections in China
Source: mSystems. 2021 Nov 2;6(6):e00837-21. doi: 10.1128/mSystems.00837-21 (PMC8562482; doi:10.1128/mSystems.00837-21)
Supplement: TABLE S1 [file msystems.00837-21-st001.docx]

**Table S1. Metadata of 749 MRSA from 2014to 2019**

| Strain | Accession | CC | ST | SCC*mec* | *spa* | Type | Year |
| --- | --- | --- | --- | --- | --- | --- | --- |
| SKLX100083 | SRR15461040 | CC59 | 59 | SCC*mec* IVa (2B) | t437 | MRSA | 2019 |
| SKLX100092 | SRR15461039 | CC59 | 59 | SCC*mec* IVa (2B) | t437 | MRSA | 2019 |
| SKLX100209 | SRR15460788 | CC59 | 5317 | SCC*mec* IVa (2B) | t437 | MRSA | 2019 |
| SKLX100269 | SRR15460901 | CC59 | 59 | SCC*mec* IVa (2B) | t437 | MRSA | 2019 |
| SKLX100316 | SRR15460734 | CC22 | 22 | SCC*mec* V (5C2&5) | t309 | MRSA | 2019 |
| SKLX100319 | SRR15460508 | CC59 | 59 | SCC*mec* V (5C2&5) | t437 | MRSA | 2019 |
| SKLX100435 | SRR15460555 | CC59 | 59 | SCC*mec* IVa (2B) | t437 | MRSA | 2019 |
| SKLX100469 | SRR15461017 | CC59 | 59 | SCC*mec* IVa (2B) | t441 | MRSA | 2019 |
| SKLX100488 | SRR15460664 | CC59 | 59 | SCC*mec* IVa (2B) | t437 | MRSA | 2019 |
| SKLX100539 | SRR15460541 | CC22 | 22 | SCC*mec* V (5C2&5) | t309 | MRSA | 2019 |
| SKLX100630 | SRR15461038 | CC59 | 59 | SCC*mec* IVa (2B) | t441 | MRSA | 2019 |
| SKLX100645 | SRR15460999 | CC59 | 59 | SCC*mec* IVa (2B) | t437 | MRSA | 2019 |
| SKLX100648 | SRR15460988 | CC59 | 59 | SCC*mec* IVa (2B) | t437 | MRSA | 2019 |
| SKLX100675 | SRR15460656 | CC59 | 59 | SCC*mec* V (5C2&5) | t437 | MRSA | 2019 |
| SKLX100707 | SRR15460645 | CC398 | 398 | SCC*mec* V (5C2) | t034 | MRSA | 2019 |
| SKLX101489 | SRR15460634 | CC22 | 22 | SCC*mec* V (5C2&5) | t309 | MRSA | 2019 |
| SKLX101499 | SRR15460493 | CC1 | 1 | Unkown | t127 | MRSA | 2019 |
| SKLX101709 | SRR15460482 | Unknown | Unknown | SCC*mec* IVa (2B) | t437 | MRSA | 2019 |
| SKLX101727 | SRR15460810 | CC5 | 5 | SCC*mec* II (2A) | t311 | HA-MRSA | 2019 |
| SKLX101735 | SRR15460799 | CC5 | 5 | SCC*mec* II (2A) | t311 | HA-MRSA | 2019 |
| SKLX102503 | SRR15460787 | CC59 | 59 | SCC*mec* V (5C2&5) | t437 | MRSA | 2019 |
| SKLX102523 | SRR15460692 | CC22 | 22 | SCC*mec* V (5C2&5) | t309 | MRSA | 2019 |
| SKLX103058 | SRR15460681 | CC398 | 398 | SCC*mec* V (5C2) | t034 | MRSA | 2019 |
| SKLX103060 | SRR15461093 | CC45 | 45 | SCC*mec* IVa (2B) | t776 | MRSA | 2019 |
| SKLX103083 | SRR15461082 | CC8 | 630 | SCC*mec* V (5C2) | t4549 | MRSA | 2019 |
| SKLX104993 | SRR15461071 | CC5 | 6 | SCC*mec* IVa (2B) | t304 | MRSA | 2019 |
| SKLX105008 | SRR15460945 | CC5 | 6 | SCC*mec* IVa (2B) | t304 | MRSA | 2019 |
| SKLX105024 | SRR15460934 | CC59 | 3355 | SCC*mec* IVa (2B) | t3523 | MRSA | 2019 |
| SKLX107050 | SRR15460923 | CC59 | 59 | SCC*mec* IVa (2B) | t437 | MRSA | 2019 |
| SKLX107203 | SRR15460912 | CC398 | 398 | SCC*mec* V (5C2) | t034 | MRSA | 2019 |
| SKLX107205 | SRR15460900 | CC398 | 398 | SCC*mec* V (5C2) | t034 | MRSA | 2019 |
| SKLX107244 | SRR15460596 | CC59 | 59 | SCC*mec* IVa (2B) | None | MRSA | 2019 |
| SKLX107301 | SRR15460585 | CC8 | 630 | SCC*mec* V (5C2&5) | None | MRSA | 2019 |
| SKLX107417 | SRR15460472 | CC59 | 59 | SCC*mec* IVa (2B) | t437 | MRSA | 2019 |
| SKLX107461 | SRR15460461 | CC59 | 59 | SCC*mec* V (5C2&5) | t437 | MRSA | 2019 |
| SKLX107516 | SRR15460450 | CC59 | 59 | SCC*mec* IVa (2B) | t437 | MRSA | 2019 |
| SKLX107637 | SRR15460439 | CC398 | 398 | SCC*mec* V (5C2) | t011 | MRSA | 2019 |
| SKLX107813 | SRR15460428 | CC59 | 338 | SCC*mec* V (5C2&5) | t437 | CA-MRSA | 2019 |
| SKLX108212 | SRR15460756 | CC59 | 4513 | SCC*mec* IVa (2B) | t437 | MRSA | 2019 |
| SKLX108216 | SRR15460745 | CC59 | 59 | SCC*mec* IVa (2B) | t441 | MRSA | 2019 |
| SKLX108240 | SRR15460733 | CC5 | 5 | SCC*mec* II (2A) | t264 | MRSA | 2019 |
| SKLX108304 | SRR15461117 | CC121 | 121 | SCC*mec* V (5C2&5) | t9518 | MRSA | 2019 |
| SKLX108426 | SRR15461106 | CC398 | 398 | SCC*mec* V (5C2) | t034 | MRSA | 2019 |
| SKLX108558 | SRR15460896 | CC59 | 59 | SCC*mec* IVa (2B) | t437 | MRSA | 2019 |
| SKLX108580 | SRR15460885 | CC59 | 59 | SCC*mec* IVa (2B) | t172 | MRSA | 2019 |
| SKLX108596 | SRR15460874 | CC59 | 59 | SCC*mec* IVa (2B) | t172 | MRSA | 2019 |
| SKLX108615 | SRR15460626 | CC22 | 22 | SCC*mec* V (5C2&5) | t309 | MRSA | 2019 |
| SKLX108650 | SRR15460615 | CC59 | 59 | SCC*mec* V (5C2&5) | t437 | MRSA | 2019 |
| SKLX108673 | SRR15460531 | CC398 | 398 | SCC*mec* V (5C2) | t011 | MRSA | 2019 |
| SKLX108693 | SRR15460519 | CC398 | 398 | SCC*mec* V (5C2) | t1255 | MRSA | 2019 |
| SKLX109209 | SRR15460507 | CC30 | 30 | SCC*mec* V (5C2&5) | t318 | MRSA | 2019 |
| SKLX109210 | SRR15460384 | CC59 | 59 | SCC*mec* IVa (2B) | t437 | MRSA | 2019 |
| SKLX109214 | SRR15460824 | CC59 | 59 | SCC*mec* IVa (2B) | t3592 | MRSA | 2019 |
| SKLX109215 | SRR15461068 | CC59 | 59 | SCC*mec* IVa (2B) | t437 | MRSA | 2019 |
| SKLX109216 | SRR15461057 | CC59 | 59 | SCC*mec* IVa (2B) | t437 | MRSA | 2019 |
| SKLX109407 | SRR15461046 | CC59 | 59 | SCC*mec* IVa (2B) | t172 | MRSA | 2019 |
| SRR109490 | SRR15460836 | CC8 | 72 | SCC*mec* IVc (2B) | t2431 | MRSA | 2019 |
| SKLX109556 | SRR15460864 | Unknown | Unknown | SCC*mec* IVa (2B) | t437 | MRSA | 2019 |
| SKLX109558 | SRR15460853 | CC59 | 59 | SCC*mec* IVa (2B) | t437 | MRSA | 2019 |
| SKLX109579 | SRR15460842 | CC59 | 59 | SCC*mec* IVa (2B) | t437 | MRSA | 2019 |
| SKLX110014 | SRR15460566 | CC59 | 59 | SCC*mec* IVa (2B) | t437 | CA-MRSA | 2019 |
| SKLX110276 | SRR15460554 | CC59 | 59 | SCC*mec* IVa (2B) | t437 | MRSA | 2019 |
| SKLX110300 | SRR15460413 | CC45 | 508 | SCC*mec* IVi (2B) | t015 | MRSA | 2019 |
| SKLX110352 | SRR15460402 | CC59 | 59 | SCC*mec* IVa (2B) | t437 | MRSA | 2019 |
| SKLX110362 | SRR15460391 | CC88 | 88 | Unkown | t6294 | MRSA | 2019 |
| SKLX111068 | SRR15460775 | CC59 | 59 | SCC*mec* IVa (2B) | None | MRSA | 2019 |
| SKLX111090 | SRR15460764 | CC22 | 22 | SCC*mec* IVj (2B) | t032 | MRSA | 2019 |
| SKLX111114 | SRR15460725 | CC5 | 5 | SCC*mec* II (2A) | t311 | MRSA | 2019 |
| SKLX111448 | SRR15460714 | CC5 | 5 | SCC*mec* II (2A) | t311 | MRSA | 2019 |
| SKLX111481 | SRR15460703 | CC59 | 59 | SCC*mec* IVa (2B) | t172 | MRSA | 2019 |
| SKLX111515 | SRR15461028 | CC5 | 5 | SCC*mec* II (2A) | t311 | MRSA | 2019 |
| SKLX111519 | SRR15461016 | CC59 | 59 | SCC*mec* IVa (2B) | t172 | CA-MRSA | 2019 |
| SKLX111531 | SRR15460977 | CC59 | 59 | SCC*mec* IVa (2B) | t172 | MRSA | 2019 |
| SKLX111539 | SRR15460966 | CC1 | 188 | SCC*mec* IVa (2B) | t189 | MRSA | 2019 |
| SKLX111548 | SRR15460955 | CC1 | 188 | SCC*mec* IVa (2B) | t189 | MRSA | 2019 |
| SKLX111553 | SRR15460832 | CC1 | 188 | SCC*mec* IVa (2B) | t189 | MRSA | 2019 |
| SKLX111584 | SRR15460669 | CC5 | 5 | SCC*mec* II (2A) | t311 | HA-MRSA | 2019 |
| SKLX112240 | SRR15460668 | CC5 | 5 | SCC*mec* II (2A) | t2460 | MRSA | 2019 |
| SKLX112632 | SRR15460667 | CC59 | 59 | SCC*mec* IVa (2B) | t16634 | MRSA | 2019 |
| SKLX112970 | SRR15460666 | CC398 | 398 | SCC*mec* V (5C2) | t034 | MRSA | 2019 |
| SKLX113385 | SRR15460665 | CC5 | 6 | SCC*mec* IVa (2B) | t304 | MRSA | 2019 |
| SKLX113386 | SRR15460663 | CC5 | 6 | SCC*mec* IVa (2B) | t304 | MRSA | 2019 |
| SKLX113775 | SRR15460662 | CC45 | 45 | SCC*mec* IVa (2B) | t1523 | MRSA | 2019 |
| SKLX113781 | SRR15460661 | CC30 | 30 | SCC*mec* IVc (2B) | t019 | MRSA | 2019 |
| SKLX113785 | SRR15460548 | CC5 | 764 | SCC*mec* II (2A) | t1084 | MRSA | 2019 |
| SKLX113866 | SRR15460547 | CC59 | 59 | SCC*mec* IVa (2B) | t437 | MRSA | 2019 |
| SKLX113971 | SRR15460546 | CC59 | 338 | SCC*mec* V (5C2&5) | t3590 | CA-MRSA | 2019 |
| SKLX114107 | SRR15460545 | CC398 | 398 | SCC*mec* V (5C2) | t034 | MRSA | 2019 |
| SKLX114126 | SRR15460544 | CC398 | 398 | SCC*mec* V (5C2) | t034 | MRSA | 2019 |
| SKLX114481 | SRR15460543 | CC5 | 5 | SCC*mec* II (2A) | t311 | MRSA | 2019 |
| SKLX114637 | SRR15460542 | CC398 | 1232 | SCC*mec* V (5C2&5) | t034 | MRSA | 2019 |
| SKLX114770 | SRR15460540 | Unknown | Unknown | SCC*mec* IVb (2B) | t309 | MRSA | 2019 |
| SKLX114776 | SRR15460539 | CC5 | 5 | SCC*mec* II (2A) | t002 | MRSA | 2019 |
| SKLX114966 | SRR15460538 | CC59 | 59 | SCC*mec* IVa (2B) | t6596 | MRSA | 2019 |
| SKLX114986 | SRR15460537 | CC59 | 59 | SCC*mec* IVa (2B) | None | MRSA | 2019 |
| SKLX115024 | SRR15460536 | CC59 | 59 | SCC*mec* IVa (2B) | t19085 | MRSA | 2019 |
| SKLX115112 | SRR15460535 | CC59 | 59 | SCC*mec* IVa (2B) | t437 | MRSA | 2019 |
| SKLX115124 | SRR15460534 | CC45 | 45 | SCC*mec* IVa (2B) | t073 | MRSA | 2019 |
| SKLX115129 | SRR15460533 | CC121 | 121 | SCC*mec* V (5C2&5) | t9518 | MRSA | 2019 |
| SKLX115137 | SRR15460532 | CC88 | 88 | SCC*mec* V (5C2&5) | t17477 | MRSA | 2019 |
| SKLX115138 | SRR15460530 | CC121 | 121 | SCC*mec* V (5C2&5) | t9518 | MRSA | 2019 |
| SKLX115141 | SRR15461009 | CC7 | 7 | SCC*mec* IVj (2B) | t091 | MRSA | 2019 |
| SKLX115178 | SRR15461008 | CC5 | 5 | SCC*mec* IVc (2B) | t010 | MRSA | 2019 |
| SRR115188 | SRR15460835 | CC8 | 72 | SCC*mec* IVc (2B) | t2431 | MRSA | 2019 |
| SRR115236 | SRR15460834 | CC8 | 72 | SCC*mec* IVc (2B) | t2431 | MRSA | 2019 |
| SKLX115288 | SRR15461007 | CC1 | 1 | SCC*mec* IVa (2B) | t4494 | MRSA | 2019 |
| SKLX115296 | SRR15461006 | CC1 | 1 | SCC*mec* IVa (2B) | t4494 | MRSA | 2019 |
| SKLX115313 | SRR15461005 | CC45 | 45 | SCC*mec* IVa (2B) | t116 | MRSA | 2019 |
| SKLX115712 | SRR15461004 | CC59 | 59 | SCC*mec* IVa (2B) | t437 | MRSA | 2019 |
| SKLX115846 | SRR15461003 | CC59 | 59 | SCC*mec* IVa (2B) | t437 | MRSA | 2019 |
| SKLX115924 | SRR15461002 | CC59 | 59 | SCC*mec* IVa (2B) | t437 | MRSA | 2019 |
| SKLX115928 | SRR15461001 | CC59 | 59 | SCC*mec* IVa (2B) | t4145 | MRSA | 2019 |
| SKLX115943 | SRR15461000 | CC8 | 239 | SCC*mec* III (3A) | t037 | MRSA | 2019 |
| SKLX115984 | SRR15460998 | CC8 | 239 | SCC*mec* III (3A) | t037 | MRSA | 2019 |
| SKLX116023 | SRR15460997 | CC5 | 5 | SCC*mec* IVc (2B) | t1560 | HA-MRSA | 2019 |
| SKLX116038 | SRR15460996 | CC5 | 965 | SCC*mec* IVc (2B) | t062 | MRSA | 2019 |
| SKLX116042 | SRR15460995 | CC45 | 508 | SCC*mec* IVi (2B) | t026 | MRSA | 2019 |
| SKLX116102 | SRR15460994 | CC59 | 59 | SCC*mec* IVa (2B) | t3424 | MRSA | 2019 |
| SKLX116114 | SRR15460993 | CC59 | 59 | SCC*mec* V (5C2&5) | t437 | MRSA | 2019 |
| SKLX116126 | SRR15460992 | CC59 | 59 | SCC*mec* IVa (2B) | t437 | MRSA | 2019 |
| SKLX116127 | SRR15460991 | CC59 | 59 | SCC*mec* IVa (2B) | t172 | MRSA | 2019 |
| SKLX116135 | SRR15460990 | CC22 | 22 | SCC*mec* V (5C2&5) | t309 | MRSA | 2019 |
| SKLX116149 | SRR15460989 | CC5 | 764 | SCC*mec* II (2A) | t002 | MRSA | 2019 |
| SKLX116294 | SRR15460987 | CC8 | 239 | SCC*mec* III (3A) | t030 | MRSA | 2019 |
| SKLX117362 | SRR15460986 | CC1 | 188 | SCC*mec* IVa (2B) | t189 | MRSA | 2019 |
| SKLX117468 | SRR15460985 | CC59 | 59 | SCC*mec* V (5C2&5) | t437 | MRSA | 2019 |
| SKLX117470 | SRR15460984 | CC5 | 965 | SCC*mec* IVc (2B) | t062 | MRSA | 2019 |
| SKLX117483 | SRR15460983 | CC59 | 59 | SCC*mec* IVg (2B) | t441 | MRSA | 2019 |
| SKLX117513 | SRR15460982 | CC59 | 59 | SCC*mec* IVa (2B) | t172 | MRSA | 2019 |
| SKLX117514 | SRR15460660 | CC30 | 30 | SCC*mec* IVc (2B) | t019 | MRSA | 2019 |
| SKLX117548 | SRR15460659 | CC59 | 3355 | SCC*mec* IVa (2B) | t437 | MRSA | 2019 |
| SKLX119012 | SRR15460658 | CC59 | 59 | SCC*mec* V (5C2&5) | t441 | MRSA | 2019 |
| SKLX119013 | SRR15460657 | CC8 | 239 | SCC*mec* III (3A) | t233 | MRSA | 2019 |
| SKLX119015 | SRR15460655 | CC59 | 59 | SCC*mec* IVa (2B) | t3523 | MRSA | 2019 |
| SKLX119044 | SRR15460654 | CC22 | 22 | SCC*mec* V (5C2&5) | t309 | MRSA | 2019 |
| SKLX119055 | SRR15460653 | CC22 | 22 | SCC*mec* V (5C2&5) | t309 | MRSA | 2019 |
| SKLX119086 | SRR15460652 | CC1 | 188 | SCC*mec* IVa (2B) | t189 | MRSA | 2019 |
| SKLX15777 | SRR15460651 | CC59 | 59 | SCC*mec* IVa (2B) | t437 | MRSA | 2014 |
| SKLX15827 | SRR15460650 | CC5 | 5 | SCC*mec* II (2A) | t311 | MRSA | 2014 |
| SKLX16270 | SRR15460649 | CC5 | 5 | SCC*mec* II (2A) | t311 | MRSA | 2014 |
| SKLX16401 | SRR15460648 | CC88 | 88 | SCC*mec* V (5C2) | t7637 | MRSA | 2014 |
| SKLX16735 | SRR15460647 | CC5 | 5 | SCC*mec* II (2A) | t311 | HA-MRSA | 2014 |
| SKLX17689 | SRR15460646 | CC5 | 5 | SCC*mec* II (2A) | t311 | HA-MRSA | 2014 |
| SKLX18135 | SRR15460644 | CC5 | 5 | SCC*mec* II (2A) | t311 | HA-MRSA | 2014 |
| SKLX18162 | SRR15460643 | CC5 | 5 | SCC*mec* II (2A) | t311 | MRSA | 2014 |
| SKLX18306 | SRR15460642 | CC5 | 5 | SCC*mec* II (2A) | t311 | HA-MRSA | 2014 |
| SKLX24210 | SRR15460641 | CC5 | 5 | SCC*mec* II (2A) | t311 | HA-MRSA | 2014 |
| SKLX24800 | SRR15460640 | CC5 | 5 | SCC*mec* II (2A) | t311 | MRSA | 2014 |
| SKLX25060 | SRR15460639 | CC8 | 630 | SCC*mec* V (5C2&5) | t4549 | MRSA | 2014 |
| SKLX25737 | SRR15460638 | CC5 | 5 | SCC*mec* II (2A) | t311 | HA-MRSA | 2014 |
| SKLX25891 | SRR15460637 | Unknown | Unknown | Unkown | t7637 | MRSA | 2014 |
| SKLX26216 | SRR15460636 | CC59 | 59 | SCC*mec* IVa (2B) | t437 | MRSA | 2014 |
| SKLX26299 | SRR15460635 | CC5 | 5 | SCC*mec* II (2A) | t002 | HA-MRSA | 2014 |
| SKLX26732 | SRR15460633 | CC5 | 5 | SCC*mec* II (2A) | t311 | HA-MRSA | 2014 |
| SKLX27323 | SRR15460502 | CC5 | 5 | SCC*mec* II (2A) | t311 | HA-MRSA | 2015 |
| SKLX27505 | SRR15460501 | CC5 | 965 | SCC*mec* IVc (2B) | t062 | MRSA | 2015 |
| SKLX27748 | SRR15460500 | CC5 | 5 | SCC*mec* II (2A) | t311 | HA-MRSA | 2015 |
| SKLX28037 | SRR15460499 | CC398 | 398 | SCC*mec* V (5C2) | t034 | MRSA | 2015 |
| SKLX28278 | SRR15460498 | CC5 | 5 | SCC*mec* II (2A) | t311 | HA-MRSA | 2015 |
| SKLX28285 | SRR15460497 | CC59 | 59 | SCC*mec* IVa (2B) | t437 | MRSA | 2015 |
| SKLX28293 | SRR15460496 | CC5 | 5 | SCC*mec* II (2A) | t311 | HA-MRSA | 2015 |
| SKLX28321 | SRR15460495 | CC5 | 5 | SCC*mec* II (2A) | t311 | MRSA | 2015 |
| SKLX28547 | SRR15460494 | CC5 | 5 | SCC*mec* II (2A) | t311 | HA-MRSA | 2015 |
| SKLX28717 | SRR15460492 | CC5 | 5 | SCC*mec* II (2A) | t311 | HA-MRSA | 2015 |
| SKLX28940 | SRR15460491 | CC5 | 5 | SCC*mec* II (2A) | t002 | MRSA | 2015 |
| SKLX29848 | SRR15460490 | CC8 | 239 | SCC*mec* III (3A) | t030 | HA-MRSA | 2015 |
| SKLX29954 | SRR15460489 | CC5 | 5 | SCC*mec* II (2A) | t311 | HA-MRSA | 2015 |
| SKLX30064 | SRR15460488 | CC5 | 5 | SCC*mec* II (2A) | t311 | HA-MRSA | 2015 |
| SKLX30455 | SRR15460487 | CC5 | 5 | SCC*mec* II (2A) | t311 | HA-MRSA | 2015 |
| SKLX30625 | SRR15460486 | CC5 | 5 | SCC*mec* II (2A) | t311 | HA-MRSA | 2015 |
| SKLX30908 | SRR15460485 | CC5 | 5 | SCC*mec* II (2A) | t311 | HA-MRSA | 2015 |
| SKLX31080 | SRR15460484 | CC59 | 3193 | SCC*mec* IVa (2B) | t172 | MRSA | 2015 |
| SKLX31081 | SRR15460483 | CC5 | 5 | SCC*mec* II (2A) | t311 | MRSA | 2015 |
| SKLX31883 | SRR15460481 | CC8 | 630 | SCC*mec* V (5C2&5) | t4549 | MRSA | 2015 |
| SKLX32421 | SRR15460480 | Unknown | Unknown | SCC*mec* IVa (2B) | t172 | MRSA | 2015 |
| SKLX32757 | SRR15460479 | CC5 | 5 | SCC*mec* II (2A) | t3557 | MRSA | 2015 |
| SKLX33447 | SRR15460478 | CC398 | 398 | SCC*mec* V (5C2) | t034 | MRSA | 2015 |
| SKLX33555 | SRR15460477 | CC5 | 5 | SCC*mec* II (2A) | t311 | HA-MRSA | 2015 |
| SKLX34055 | SRR15460476 | CC5 | 5 | SCC*mec* II (2A) | t311 | HA-MRSA | 2015 |
| SKLX34646 | SRR15460475 | CC5 | 5 | SCC*mec* II (2A) | t311 | HA-MRSA | 2015 |
| SKLX34951 | SRR15460813 | CC398 | 398 | SCC*mec* V (5C2) | t034 | MRSA | 2015 |
| SKLX35241 | SRR15460812 | CC5 | 5 | SCC*mec* II (2A) | t311 | MRSA | 2015 |
| SKLX35874 | SRR15460811 | CC5 | 5 | SCC*mec* II (2A) | t311 | HA-MRSA | 2015 |
| SKLX36296 | SRR15460809 | CC5 | 5 | SCC*mec* II (2A) | t311 | HA-MRSA | 2016 |
| SKLX36764 | SRR15460808 | CC59 | 338 | SCC*mec* V (5C2&5) | t13774 | CA-MRSA | 2016 |
| SKLX37159 | SRR15460807 | CC5 | 5 | SCC*mec* II (2A) | t311 | HA-MRSA | 2016 |
| SKLX37344 | SRR15460806 | CC59 | 59 | SCC*mec* IVa (2B) | t437 | MRSA | 2016 |
| SKLX37345 | SRR15460805 | CC59 | 59 | SCC*mec* IVa (2B) | t437 | MRSA | 2016 |
| SKLX37716 | SRR15460804 | CC398 | 398 | SCC*mec* V (5C2) | t034 | MRSA | 2016 |
| SKLX37803 | SRR15460803 | CC59 | 59 | SCC*mec* IVa (2B) | t437 | CA-MRSA | 2016 |
| SKLX37808 | SRR15460802 | CC5 | 5 | SCC*mec* II (2A) | t311 | HA-MRSA | 2016 |
| SKLX37885 | SRR15460801 | CC5 | 5 | SCC*mec* II (2A) | t311 | MRSA | 2016 |
| SKLX38043 | SRR15460800 | CC5 | 5 | SCC*mec* II (2A) | t311 | MRSA | 2016 |
| SKLX38960 | SRR15460798 | CC5 | 5 | SCC*mec* II (2A) | t311 | HA-MRSA | 2016 |
| SKLX39166 | SRR15460797 | CC8 | 239 | SCC*mec* III (3A) | t421 | MRSA | 2016 |
| SKLX39359 | SRR15460796 | CC5 | 5 | SCC*mec* II (2A) | t311 | MRSA | 2016 |
| SKLX39449 | SRR15460795 | CC5 | 764 | SCC*mec* II (2A) | t045 | MRSA | 2016 |
| SKLX39485 | SRR15460794 | CC59 | 59 | SCC*mec* IVa (2B) | t437 | MRSA | 2016 |
| SKLX39989 | SRR15460793 | CC5 | 5 | SCC*mec* II (2A) | t2731 | MRSA | 2016 |
| SKLX40085 | SRR15460792 | CC5 | 5 | SCC*mec* II (2A) | t311 | HA-MRSA | 2016 |
| SKLX40627 | SRR15460791 | CC5 | 5 | SCC*mec* II (2A) | t311 | MRSA | 2016 |
| SKLX40823 | SRR15460790 | CC5 | 5 | SCC*mec* II (2A) | t311 | HA-MRSA | 2016 |
| SKLX41515 | SRR15460789 | CC59 | 59 | SCC*mec* IVa (2B) | t163 | MRSA | 2016 |
| SKLX41869 | SRR15460786 | CC5 | 5 | SCC*mec* II (2A) | t311 | MRSA | 2016 |
| SKLX42090 | SRR15460701 | CC59 | 59 | SCC*mec* IVa (2B) | t163 | MRSA | 2016 |
| SKLX43302 | SRR15460700 | Unknown | Unknown | SCC*mec* II (2A) | t311 | MRSA | 2016 |
| SKLX43765 | SRR15460699 | CC1 | 188 | SCC*mec* IVa (2B) | t3887 | MRSA | 2016 |
| SKLX45006 | SRR15460698 | CC59 | 59 | SCC*mec* IVa (2B) | t437 | CA-MRSA | 2017 |
| SKLX45729 | SRR15460697 | CC5 | 5 | SCC*mec* II (2A) | t311 | MRSA | 2017 |
| SKLX46857 | SRR15460696 | CC5 | 5 | SCC*mec* II (2A) | t311 | MRSA | 2017 |
| SKLX47505 | SRR15460695 | CC5 | 5 | SCC*mec* II (2A) | t311 | MRSA | 2017 |
| SKLX47820 | SRR15460694 | CC5 | 5 | SCC*mec* II (2A) | t311 | HA-MRSA | 2017 |
| SKLX47979 | SRR15460693 | CC5 | 5 | SCC*mec* II (2A) | t311 | HA-MRSA | 2017 |
| SKLX48445 | SRR15460691 | Unknown | Unknown | SCC*mec* IVa (2B) | t693 | MRSA | 2017 |
| SKLX48519 | SRR15460690 | CC5 | 5 | SCC*mec* II (2A) | t311 | HA-MRSA | 2017 |
| SKLX49750 | SRR15460689 | CC398 | 398 | SCC*mec* V (5C2) | t034 | MRSA | 2017 |
| SKLX50248 | SRR15460688 | CC5 | 5 | SCC*mec* II (2A) | t311 | MRSA | 2014 |
| SKLX50258 | SRR15460687 | CC5 | 5 | SCC*mec* II (2A) | t311 | MRSA | 2014 |
| SKLX50298 | SRR15460686 | CC5 | 5 | SCC*mec* II (2A) | t311 | MRSA | 2014 |
| SKLX50445 | SRR15460685 | CC88 | 88 | SCC*mec* IVa (2B) | t3622 | MRSA | 2014 |
| SKLX50517 | SRR15460684 | Unknown | Unknown | SCC*mec* III (3A) | t030 | MRSA | 2014 |
| SKLX50577 | SRR15460683 | CC59 | 59 | SCC*mec* IVa (2B) | t163 | MRSA | 2014 |
| SKLX50636 | SRR15460682 | CC59 | 338 | SCC*mec* V (5C2&5) | t437 | MRSA | 2014 |
| SKLX50637 | SRR15460680 | CC5 | 5 | SCC*mec* II (2A) | t002 | MRSA | 2014 |
| SKLX50712 | SRR15460679 | CC59 | 59 | SCC*mec* IVa (2B) | t172 | MRSA | 2014 |
| SKLX50714 | SRR15460678 | CC88 | 88 | SCC*mec* IVc (2B) | t2310 | MRSA | 2014 |
| SKLX50717 | SRR15460677 | CC59 | 59 | SCC*mec* IVa (2B) | t172 | MRSA | 2014 |
| SKLX50775 | SRR15460676 | CC1 | 1 | SCC*mec* IVc (2B) | t1508 | MRSA | 2014 |
| SKLX50788 | SRR15460675 | CC59 | 59 | SCC*mec* IVa (2B) | t441 | MRSA | 2014 |
| SKLX50832 | SRR15460674 | CC88 | 88 | Unkown | t7637 | MRSA | 2014 |
| SKLX50938 | SRR15461096 | CC59 | 59 | SCC*mec* IVa (2B) | t437 | MRSA | 2014 |
| SKLX50940 | SRR15461095 | CC59 | 59 | SCC*mec* IVa (2B) | t1151 | MRSA | 2014 |
| SKLX50944 | SRR15461094 | CC8 | 239 | SCC*mec* III (3A) | t030 | MRSA | 2014 |
| SKLX51063 | SRR15461092 | CC59 | 59 | SCC*mec* IVa (2B) | t437 | MRSA | 2014 |
| SKLX51104 | SRR15461091 | CC8 | 239 | SCC*mec* III (3A) | t030 | MRSA | 2014 |
| SKLX51240 | SRR15461090 | CC8 | 239 | SCC*mec* III (3A) | t030 | MRSA | 2014 |
| SKLX51312 | SRR15461089 | CC5 | 5 | SCC*mec* II (2A) | t002 | HA-MRSA | 2014 |
| SKLX51378 | SRR15461088 | CC5 | 5 | SCC*mec* II (2A) | t311 | HA-MRSA | 2014 |
| SKLX51389 | SRR15461087 | CC5 | 5 | SCC*mec* II (2A) | t311 | MRSA | 2014 |
| SKLX51393 | SRR15461086 | CC5 | 5 | SCC*mec* II (2A) | t311 | MRSA | 2014 |
| SKLX51395 | SRR15461085 | CC5 | 5 | SCC*mec* II (2A) | t311 | MRSA | 2014 |
| SKLX51408 | SRR15461084 | CC5 | 5 | SCC*mec* II (2A) | t311 | MRSA | 2014 |
| SKLX51492 | SRR15461083 | CC59 | 59 | SCC*mec* V (5C2&5) | t437 | MRSA | 2014 |
| SKLX51493 | SRR15461081 | CC59 | 59 | SCC*mec* IVa (2B) | t437 | MRSA | 2014 |
| SKLX51620 | SRR15461080 | CC45 | 45 | SCC*mec* IVa (2B) | t116 | MRSA | 2014 |
| SKLX51654 | SRR15461079 | CC5 | 5 | SCC*mec* II (2A) | t311 | HA-MRSA | 2014 |
| SKLX51666 | SRR15461078 | CC5 | 5 | SCC*mec* II (2A) | t311 | MRSA | 2015 |
| SKLX51683 | SRR15461077 | CC1 | 1 | SCC*mec* IVg (2B) | t114 | MRSA | 2014 |
| SKLX51707 | SRR15461076 | CC5 | 965 | SCC*mec* IVc (2B) | t062 | MRSA | 2014 |
| SKLX51784 | SRR15461075 | CC59 | 59 | SCC*mec* IVa (2B) | t437 | MRSA | 2014 |
| SKLX51793 | SRR15461074 | CC59 | 59 | SCC*mec* V (5C2&5) | t437 | MRSA | 2014 |
| SKLX51800 | SRR15461073 | CC88 | 88 | Unkown | t10793 | MRSA | 2014 |
| SKLX51867 | SRR15461072 | CC5 | 5 | SCC*mec* II (2A) | t311 | MRSA | 2014 |
| SKLX51897 | SRR15461070 | CC59 | 59 | SCC*mec* IVa (2B) | t437 | MRSA | 2014 |
| SKLX52177 | SRR15461069 | CC5 | 5 | SCC*mec* II (2A) | t002 | MRSA | 2014 |
| SKLX52179 | SRR15460953 | CC88 | 88 | Unkown | None | MRSA | 2014 |
| SKLX52230 | SRR15460952 | CC8 | 239 | SCC*mec* III (3A) | t030 | MRSA | 2014 |
| SKLX52232 | SRR15460951 | CC398 | 398 | SCC*mec* V (5C2) | t034 | MRSA | 2014 |
| SKLX52320 | SRR15460950 | CC8 | 239 | SCC*mec* III (3A) | t030 | MRSA | 2014 |
| SKLX52339 | SRR15460949 | CC8 | 239 | SCC*mec* III (3A) | t030 | MRSA | 2014 |
| SKLX52399 | SRR15460948 | CC30 | 30 | SCC*mec* IVa (2B) | t2147 | MRSA | 2014 |
| SKLX52512 | SRR15460947 | CC59 | 59 | SCC*mec* IVa (2B) | t437 | MRSA | 2014 |
| SKLX52612 | SRR15460946 | CC8 | 239 | SCC*mec* III (3A) | t030 | MRSA | 2014 |
| SKLX52742 | SRR15460944 | CC59 | 59 | SCC*mec* IVa (2B) | t163 | MRSA | 2014 |
| SKLX52805 | SRR15460943 | CC398 | 5539 | SCC*mec* V (5C2) | t034 | MRSA | 2014 |
| SKLX52838 | SRR15460942 | CC5 | 5 | SCC*mec* II (2A) | t2460 | HA-MRSA | 2014 |
| SKLX52851 | SRR15460941 | CC59 | 59 | SCC*mec* IVa (2B) | t172 | MRSA | 2014 |
| SKLX52855 | SRR15460940 | CC5 | 5 | SCC*mec* II (2A) | t2460 | HA-MRSA | 2014 |
| SKLX52880 | SRR15460939 | CC398 | 398 | SCC*mec* V (5C2) | t034 | MRSA | 2015 |
| SKLX52911 | SRR15460938 | CC59 | 59 | SCC*mec* IVa (2B) | t437 | MRSA | 2015 |
| SKLX52922 | SRR15460937 | CC5 | 5 | SCC*mec* II (2A) | t311 | HA-MRSA | 2015 |
| SKLX53001 | SRR15460936 | CC59 | 338 | SCC*mec* V (5C2&5) | t437 | CA-MRSA | 2015 |
| SKLX53010 | SRR15460935 | CC59 | 59 | SCC*mec* IVa (2B) | t437 | MRSA | 2015 |
| SKLX53030 | SRR15460933 | CC59 | 59 | SCC*mec* IVa (2B) | t437 | MRSA | 2015 |
| SKLX53068 | SRR15460932 | CC59 | 59 | SCC*mec* IVa (2B) | None | MRSA | 2015 |
| SKLX53231 | SRR15460931 | CC5 | 5 | SCC*mec* II (2A) | t311 | MRSA | 2015 |
| SKLX53288 | SRR15460930 | CC5 | 5 | SCC*mec* II (2A) | t311 | MRSA | 2015 |
| SKLX53308 | SRR15460929 | CC5 | 5 | SCC*mec* II (2A) | t002 | HA-MRSA | 2015 |
| SKLX53321 | SRR15460928 | CC59 | 59 | SCC*mec* IVa (2B) | t3523 | CA-MRSA | 2015 |
| SKLX53342 | SRR15460927 | CC5 | 5 | SCC*mec* II (2A) | t311 | MRSA | 2015 |
| SKLX53357 | SRR15460926 | CC5 | 5 | SCC*mec* II (2A) | t311 | MRSA | 2015 |
| SKLX53388 | SRR15460925 | CC5 | 5 | SCC*mec* II (2A) | t311 | HA-MRSA | 2015 |
| SKLX53417 | SRR15460924 | CC8 | 239 | SCC*mec* III (3A) | t037 | HA-MRSA | 2015 |
| SKLX53430 | SRR15460922 | Unknown | Unknown | SCC*mec* II (2A) | t002 | MRSA | 2015 |
| SKLX53440 | SRR15460921 | CC5 | 5 | SCC*mec* II (2A) | t002 | MRSA | 2015 |
| SKLX53474 | SRR15460920 | CC398 | 5539 | SCC*mec* V (5C2) | t034 | MRSA | 2015 |
| SKLX53476 | SRR15460919 | CC59 | 59 | SCC*mec* IVa (2B) | t437 | MRSA | 2015 |
| SKLX53477 | SRR15460918 | CC5 | 5529 | SCC*mec* IVg (2B) | t688 | MRSA | 2015 |
| SKLX53478 | SRR15460917 | CC59 | 59 | SCC*mec* IVa (2B) | t437 | MRSA | 2015 |
| SKLX53479 | SRR15460916 | CC59 | 59 | SCC*mec* V (5C2&5) | t437 | MRSA | 2015 |
| SKLX53480 | SRR15460915 | CC5 | 5 | SCC*mec* II (2A) | t002 | MRSA | 2015 |
| SKLX53484 | SRR15460914 | CC59 | 59 | SCC*mec* IVa (2B) | t163 | MRSA | 2015 |
| SKLX53487 | SRR15460913 | CC59 | 59 | SCC*mec* IVa (2B) | t437 | MRSA | 2015 |
| SKLX53534 | SRR15460911 | CC59 | 59 | SCC*mec* IVa (2B) | t172 | MRSA | 2015 |
| SKLX53577 | SRR15460910 | CC59 | 59 | SCC*mec* IVa (2B) | t172 | MRSA | 2015 |
| SKLX53588 | SRR15460909 | CC8 | 239 | SCC*mec* III (3A) | t030 | MRSA | 2015 |
| SKLX53611 | SRR15460908 | CC59 | 59 | SCC*mec* IVa (2B) | t437 | MRSA | 2015 |
| SKLX53655 | SRR15460907 | CC8 | 630 | SCC*mec* V (5C2&5) | t4549 | MRSA | 2015 |
| SKLX53755 | SRR15460906 | CC59 | 59 | SCC*mec* IVg (2B) | t1751 | MRSA | 2015 |
| SKLX53781 | SRR15460905 | CC59 | 59 | SCC*mec* IVg (2B) | t1751 | MRSA | 2015 |
| SKLX53867 | SRR15460904 | CC59 | 59 | SCC*mec* IVa (2B) | t437 | MRSA | 2015 |
| SKLX53973 | SRR15460903 | CC59 | 59 | SCC*mec* IVa (2B) | t437 | MRSA | 2015 |
| SKLX53974 | SRR15460902 | CC59 | 59 | SCC*mec* IVa (2B) | t437 | MRSA | 2015 |
| SKLX54052 | SRR15460899 | CC59 | 59 | SCC*mec* IVa (2B) | t437 | MRSA | 2015 |
| SKLX54055 | SRR15460898 | CC121 | 121 | SCC*mec* V (5C2&5) | t8660 | MRSA | 2015 |
| SKLX54114 | SRR15460604 | CC5 | 5 | SCC*mec* II (2A) | t311 | HA-MRSA | 2015 |
| SKLX54120 | SRR15460603 | CC8 | 239 | SCC*mec* III (3A) | t030 | MRSA | 2015 |
| SKLX54127 | SRR15460602 | CC5 | 764 | SCC*mec* II (2A) | t002 | MRSA | 2015 |
| SKLX54236 | SRR15460601 | CC5 | 5 | SCC*mec* II (2A) | t311 | MRSA | 2015 |
| SKLX54268 | SRR15460600 | CC5 | 5 | SCC*mec* II (2A) | t311 | HA-MRSA | 2015 |
| SKLX54320 | SRR15460599 | CC8 | 239 | SCC*mec* III (3A) | t030 | MRSA | 2015 |
| SKLX54431 | SRR15460598 | CC59 | 59 | SCC*mec* IVa (2B) | t437 | MRSA | 2015 |
| SKLX54459 | SRR15460597 | CC59 | 59 | SCC*mec* IVa (2B) | t3424 | MRSA | 2015 |
| SKLX54479 | SRR15460595 | CC59 | 59 | SCC*mec* IVa (2B) | t437 | MRSA | 2015 |
| SKLX54561 | SRR15460594 | CC8 | 239 | SCC*mec* III (3A) | t632 | MRSA | 2015 |
| SKLX54590 | SRR15460593 | CC59 | 59 | SCC*mec* IVa (2B) | t519 | MRSA | 2015 |
| SKLX54652 | SRR15460592 | CC8 | 239 | SCC*mec* III (3A) | t037 | MRSA | 2015 |
| SKLX54693 | SRR15460591 | CC59 | 59 | SCC*mec* IVa (2B) | t441 | MRSA | 2015 |
| SKLX54694 | SRR15460590 | CC45 | 508 | SCC*mec* IVi (2B) | t015 | MRSA | 2015 |
| SKLX54695 | SRR15460589 | CC59 | 59 | SCC*mec* V (5C2&5) | t437 | MRSA | 2015 |
| SKLX54710 | SRR15460588 | CC8 | 630 | SCC*mec* V (5C2) | t4549 | MRSA | 2015 |
| SKLX54720 | SRR15460587 | CC59 | 59 | SCC*mec* IVa (2B) | t437 | MRSA | 2015 |
| SKLX54735 | SRR15460586 | CC45 | 508 | SCC*mec* IVi (2B) | t1608 | MRSA | 2015 |
| SKLX54743 | SRR15460584 | CC59 | 59 | SCC*mec* IVg (2B) | t1751 | MRSA | 2015 |
| SKLX54749 | SRR15460583 | CC59 | 59 | SCC*mec* V (5C2&5) | t437 | MRSA | 2015 |
| SKLX54750 | SRR15460582 | CC59 | 59 | SCC*mec* IVa (2B) | t437 | MRSA | 2015 |
| SKLX54751 | SRR15460581 | CC59 | 59 | SCC*mec* IVg (2B) | t9460 | MRSA | 2015 |
| SKLX54839 | SRR15460580 | Unknown | Unknown | SCC*mec* IVa (2B) | t8296 | MRSA | 2015 |
| SKLX55037 | SRR15460579 | CC5 | 5 | SCC*mec* II (2A) | t002 | MRSA | 2015 |
| SKLX55040 | SRR15460578 | CC59 | 59 | SCC*mec* IVa (2B) | t437 | MRSA | 2015 |
| SKLX55046 | SRR15460577 | CC59 | 59 | SCC*mec* IVa (2B) | t172 | CA-MRSA | 2015 |
| SKLX55077 | SRR15460474 | CC5 | 5 | SCC*mec* II (2A) | t311 | MRSA | 2015 |
| SKLX55132 | SRR15460473 | CC30 | 2580 | SCC*mec* IVc (2B) | t3351 | MRSA | 2015 |
| SKLX55133 | SRR15460471 | CC30 | 2580 | SCC*mec* IVc (2B) | t3351 | MRSA | 2015 |
| SKLX55182 | SRR15460470 | CC5 | 5 | SCC*mec* II (2A) | t311 | HA-MRSA | 2015 |
| SKLX55190 | SRR15460469 | CC5 | 5 | SCC*mec* II (2A) | t311 | HA-MRSA | 2015 |
| SKLX55351 | SRR15460468 | CC59 | 59 | SCC*mec* IVa (2B) | t437 | MRSA | 2015 |
| SKLX55383 | SRR15460467 | CC59 | 4513 | SCC*mec* IVa (2B) | t437 | MRSA | 2016 |
| SKLX55416 | SRR15460466 | CC59 | 59 | SCC*mec* V (5C2&5) | t437 | MRSA | 2016 |
| SKLX55421 | SRR15460465 | CC59 | 59 | SCC*mec* V (5C2&5) | t437 | MRSA | 2016 |
| SKLX55422 | SRR15460464 | Unknown | Unknown | SCC*mec* V (5C2&5) | t045 | MRSA | 2016 |
| SKLX55486 | SRR15460463 | CC59 | 59 | SCC*mec* IVa (2B) | t441 | MRSA | 2016 |
| SKLX55499 | SRR15460462 | CC59 | 59 | SCC*mec* IVa (2B) | t441 | MRSA | 2016 |
| SKLX55521 | SRR15460460 | CC59 | 59 | SCC*mec* V (5C2&5) | t437 | MRSA | 2016 |
| SKLX55539 | SRR15460459 | CC45 | 508 | SCC*mec* IVi (2B) | t1608 | MRSA | 2016 |
| SKLX55554 | SRR15460458 | CC59 | 59 | SCC*mec* IVa (2B) | t441 | MRSA | 2016 |
| SKLX55555 | SRR15460457 | CC5 | 965 | SCC*mec* IVc (2B) | t062 | MRSA | 2016 |
| SKLX55622 | SRR15460456 | CC5 | 5 | SCC*mec* II (2A) | t311 | MRSA | 2016 |
| SKLX55638 | SRR15460455 | CC59 | 59 | SCC*mec* IVa (2B) | t172 | MRSA | 2015 |
| SRR55661 | SRR15460833 | CC8 | 72 | SCC*mec* IVc (2B) | t2461 | MRSA | 2015 |
| SKLX55793 | SRR15460454 | CC398 | 3681 | Unkown | t1255 | MRSA | 2015 |
| SKLX55795 | SRR15460453 | CC398 | 3681 | Unkown | t1255 | MRSA | 2015 |
| SKLX55797 | SRR15460452 | CC59 | 59 | SCC*mec* IVa (2B) | t437 | MRSA | 2015 |
| SKLX56027 | SRR15460451 | CC59 | 3355 | SCC*mec* IVa (2B) | t437 | MRSA | 2015 |
| SKLX56053 | SRR15460449 | CC8 | 239 | SCC*mec* III (3A) | t030 | MRSA | 2015 |
| SKLX56176 | SRR15460448 | CC7 | 7 | SCC*mec* V (5C2&5) | t796 | MRSA | 2016 |
| SKLX56187 | SRR15460447 | Unknown | Unknown | SCC*mec* V (5C2) | t1764 | MRSA | 2016 |
| SKLX56193 | SRR15460446 | CC8 | 239 | SCC*mec* III (3A) | t030 | MRSA | 2016 |
| SKLX56215 | SRR15460445 | CC59 | 4513 | SCC*mec* IVa (2B) | t441 | MRSA | 2016 |
| SKLX56216 | SRR15460444 | CC8 | 239 | SCC*mec* III (3A) | t030 | MRSA | 2016 |
| SKLX56222 | SRR15460443 | CC1 | 1 | SCC*mec* IVg (2B) | t114 | MRSA | 2016 |
| SKLX56231 | SRR15460442 | CC5 | 5 | SCC*mec* II (2A) | t17573 | MRSA | 2016 |
| SKLX56262 | SRR15460441 | CC1 | 1 | SCC*mec* IVa (2B) | t1908 | MRSA | 2016 |
| SKLX56266 | SRR15460440 | Unknown | Unknown | SCC*mec* II (2A) | None | MRSA | 2016 |
| SKLX56296 | SRR15460438 | CC8 | 239 | SCC*mec* III (3A) | t037 | MRSA | 2016 |
| SKLX56302 | SRR15460437 | CC59 | 59 | SCC*mec* V (5C2&5) | t437 | MRSA | 2016 |
| SKLX56310 | SRR15460436 | CC398 | 398 | SCC*mec* V (5C2) | t034 | MRSA | 2016 |
| SKLX56367 | SRR15460435 | CC59 | 59 | SCC*mec* IVa (2B) | t172 | MRSA | 2016 |
| SKLX56513 | SRR15460434 | CC59 | 59 | SCC*mec* IVa (2B) | t437 | MRSA | 2016 |
| SKLX56569 | SRR15460433 | CC59 | 59 | SCC*mec* IVa (2B) | t437 | MRSA | 2016 |
| SKLX56578 | SRR15460432 | CC59 | 59 | SCC*mec* IVg (2B) | t1751 | MRSA | 2016 |
| SKLX56579 | SRR15460431 | CC59 | 59 | SCC*mec* IVa (2B) | t3424 | MRSA | 2016 |
| SKLX56588 | SRR15460430 | CC59 | 59 | SCC*mec* IVg (2B) | t1751 | MRSA | 2016 |
| SKLX56590 | SRR15460429 | CC59 | 59 | SCC*mec* V (5C2&5) | t437 | MRSA | 2016 |
| SKLX56593 | SRR15460427 | CC121 | 121 | SCC*mec* V (5C2&5) | t8660 | MRSA | 2016 |
| SKLX56609 | SRR15460426 | CC59 | 338 | SCC*mec* V (5C2&5) | t437 | CA-MRSA | 2016 |
| SKLX56713 | SRR15460425 | CC8 | 239 | SCC*mec* III (3A) | t030 | MRSA | 2016 |
| SRR56774 | SRR15460831 | CC8 | 72 | SCC*mec* V (5C2&5) | t148 | MRSA | 2016 |
| SRR56776 | SRR15460830 | CC8 | 72 | SCC*mec* V (5C2&5) | t148 | MRSA | 2016 |
| SKLX56841 | SRR15460424 | CC5 | 5 | SCC*mec* II (2A) | t311 | HA-MRSA | 2016 |
| SKLX56848 | SRR15460423 | CC5 | 5 | SCC*mec* II (2A) | t311 | HA-MRSA | 2016 |
| SRR56854 | SRR15460829 | CC8 | 72 | SCC*mec* V (5C2&5) | t4100 | MRSA | 2016 |
| SKLX56885 | SRR15460422 | CC5 | 5 | SCC*mec* II (2A) | t311 | HA-MRSA | 2016 |
| SKLX56945 | SRR15460421 | CC59 | 59 | SCC*mec* IVa (2B) | t437 | MRSA | 2016 |
| SKLX56991 | SRR15460420 | CC59 | 5525 | SCC*mec* IVa (2B) | t437 | MRSA | 2016 |
| SKLX56993 | SRR15460419 | CC59 | 5525 | SCC*mec* IVa (2B) | t437 | MRSA | 2016 |
| SKLX57137 | SRR15460757 | CC59 | 59 | SCC*mec* IVa (2B) | t163 | MRSA | 2016 |
| SKLX57229 | SRR15460755 | CC59 | 59 | SCC*mec* IVa (2B) | t437 | MRSA | 2016 |
| SKLX57230 | SRR15460754 | CC59 | 59 | SCC*mec* IVa (2B) | t437 | MRSA | 2016 |
| SKLX57233 | SRR15460753 | CC59 | 59 | SCC*mec* IVa (2B) | t437 | MRSA | 2017 |
| SKLX57234 | SRR15460752 | CC59 | 59 | SCC*mec* IVa (2B) | t437 | MRSA | 2016 |
| SKLX57237 | SRR15460751 | CC398 | 398 | SCC*mec* V (5C2) | t034 | MRSA | 2016 |
| SKLX57238 | SRR15460750 | CC398 | 398 | SCC*mec* V (5C2) | t034 | MRSA | 2016 |
| SKLX57249 | SRR15460749 | CC59 | 59 | SCC*mec* IVa (2B) | t437 | MRSA | 2016 |
| SKLX57252 | SRR15460748 | CC59 | 59 | SCC*mec* IVa (2B) | t437 | MRSA | 2016 |
| SKLX57282 | SRR15460747 | CC59 | 338 | SCC*mec* V (5C2&5) | t437 | CA-MRSA | 2016 |
| SKLX57323 | SRR15460746 | CC59 | 59 | SCC*mec* IVa (2B) | t172 | MRSA | 2016 |
| SKLX57330 | SRR15460744 | CC59 | 59 | SCC*mec* IVa (2B) | t441 | MRSA | 2016 |
| SKLX57501 | SRR15460743 | Unknown | Unknown | SCC*mec* IVa (2B) | t441 | MRSA | 2016 |
| SKLX57645 | SRR15460742 | CC59 | 59 | SCC*mec* IVa (2B) | t437 | MRSA | 2016 |
| SKLX57650 | SRR15460741 | CC45 | 508 | SCC*mec* IVi (2B) | t1608 | MRSA | 2016 |
| SKLX57651 | SRR15460740 | CC59 | 59 | SCC*mec* IVg (2B) | t437 | MRSA | 2016 |
| SKLX57656 | SRR15460739 | CC59 | 59 | SCC*mec* IVg (2B) | t437 | MRSA | 2016 |
| SKLX57689 | SRR15460738 | CC59 | 59 | SCC*mec* IVa (2B) | t437 | MRSA | 2016 |
| SKLX57696 | SRR15460737 | CC59 | 59 | SCC*mec* IVa (2B) | t3385 | MRSA | 2016 |
| SKLX57701 | SRR15460736 | CC88 | 88 | SCC*mec* V (5C2) | t12147 | MRSA | 2016 |
| SKLX57702 | SRR15460735 | CC59 | 59 | SCC*mec* IVa (2B) | t3385 | MRSA | 2016 |
| SKLX57794 | SRR15460732 | CC59 | 5525 | SCC*mec* IVa (2B) | t437 | MRSA | 2016 |
| SKLX57801 | SRR15460731 | CC8 | 630 | SCC*mec* V (5C2&5) | t4549 | MRSA | 2016 |
| SKLX57866 | SRR15460730 | CC59 | 59 | SCC*mec* IVa (2B) | t437 | MRSA | 2016 |
| SKLX57946 | SRR15460827 | CC59 | 59 | SCC*mec* IVa (2B) | t437 | MRSA | 2016 |
| SKLX57947 | SRR15461123 | CC1 | 1 | SCC*mec* IVg (2B) | t114 | MRSA | 2016 |
| SKLX58002 | SRR15461122 | CC59 | 59 | SCC*mec* V (5C2&5) | t437 | MRSA | 2016 |
| SKLX58004 | SRR15461121 | CC59 | 59 | SCC*mec* V (5C2&5) | t437 | MRSA | 2016 |
| SKLX58009 | SRR15461120 | CC8 | 239 | SCC*mec* III (3A) | t030 | MRSA | 2016 |
| SKLX58095 | SRR15461119 | CC8 | 239 | SCC*mec* III (3A) | t030 | MRSA | 2016 |
| SKLX58116 | SRR15461118 | CC8 | 239 | SCC*mec* III (3A) | t030 | MRSA | 2016 |
| SKLX58137 | SRR15461116 | CC59 | 59 | SCC*mec* IVa (2B) | t437 | MRSA | 2016 |
| SKLX58278 | SRR15461115 | CC398 | 398 | SCC*mec* V (5C2) | t4652 | MRSA | 2016 |
| SKLX58283 | SRR15461114 | CC5 | 764 | SCC*mec* II (2A) | t002 | MRSA | 2016 |
| SKLX58350 | SRR15461113 | CC59 | 59 | SCC*mec* IVa (2B) | t437 | MRSA | 2016 |
| SKLX58351 | SRR15461112 | CC59 | 338 | SCC*mec* V (5C2&5) | t437 | CA-MRSA | 2016 |
| SKLX58368 | SRR15461111 | CC88 | 88 | SCC*mec* IVc (2B) | t2310 | MRSA | 2016 |
| SRR58369 | SRR15460828 | CC8 | 72 | SCC*mec* IVc (2B) | t664 | MRSA | 2016 |
| SKLX58663 | SRR15461110 | CC88 | 88 | SCC*mec* IVc (2B) | None | MRSA | 2016 |
| SKLX58708 | SRR15461109 | CC59 | 59 | SCC*mec* IVa (2B) | t437 | MRSA | 2016 |
| SKLX58711 | SRR15461108 | CC59 | 59 | SCC*mec* IVa (2B) | t437 | MRSA | 2016 |
| SKLX58725 | SRR15461107 | CC59 | 59 | SCC*mec* V (5C2&5) | t437 | MRSA | 2016 |
| SKLX58740 | SRR15461105 | CC5 | 965 | SCC*mec* IVc (2B) | t062 | MRSA | 2016 |
| SKLX58741 | SRR15461104 | CC5 | 5 | SCC*mec* II (2A) | t311 | MRSA | 2016 |
| SKLX58752 | SRR15461103 | CC59 | 4513 | SCC*mec* IVa (2B) | t8886 | MRSA | 2016 |
| SKLX58763 | SRR15461102 | CC5 | 965 | SCC*mec* IVc (2B) | t062 | MRSA | 2016 |
| SKLX58799 | SRR15461101 | CC5 | 5 | SCC*mec* II (2A) | t311 | HA-MRSA | 2016 |
| SKLX58815 | SRR15461100 | CC5 | 5 | SCC*mec* II (2A) | t311 | MRSA | 2016 |
| SKLX58897 | SRR15461099 | CC59 | 59 | SCC*mec* IVa (2B) | t437 | MRSA | 2016 |
| SKLX58898 | SRR15461098 | CC59 | 59 | SCC*mec* IVa (2B) | t437 | MRSA | 2016 |
| SKLX58947 | SRR15461097 | Unknown | Unknown | SCC*mec* IVa (2B) | t3523 | MRSA | 2016 |
| SKLX58958 | SRR15460897 | Unknown | Unknown | SCC*mec* V (5C2&5) | t1751 | MRSA | 2016 |
| SKLX58971 | SRR15460895 | CC8 | 239 | SCC*mec* III (3A) | t030 | MRSA | 2016 |
| SKLX59015 | SRR15460894 | CC8 | 239 | SCC*mec* III (3A) | t030 | MRSA | 2016 |
| SKLX59048 | SRR15460893 | CC8 | 239 | SCC*mec* III (3A) | t030 | MRSA | 2016 |
| SKLX59080 | SRR15460892 | CC59 | 59 | SCC*mec* IVa (2B) | t437 | MRSA | 2016 |
| SKLX59234 | SRR15460891 | CC8 | 239 | SCC*mec* III (3A) | t030 | MRSA | 2016 |
| SKLX59235 | SRR15460890 | CC8 | 239 | SCC*mec* III (3A) | t030 | MRSA | 2016 |
| SKLX59236 | SRR15460889 | CC8 | 239 | SCC*mec* III (3A) | t030 | MRSA | 2016 |
| SKLX59237 | SRR15460888 | CC8 | 239 | SCC*mec* III (3A) | t030 | MRSA | 2016 |
| SKLX59279 | SRR15460887 | CC59 | 59 | SCC*mec* IVa (2B) | t519 | MRSA | 2016 |
| SKLX59280 | SRR15460886 | CC59 | 59 | SCC*mec* IVa (2B) | t519 | MRSA | 2016 |
| SKLX59393 | SRR15460884 | CC59 | 59 | SCC*mec* IVa (2B) | t437 | MRSA | 2016 |
| SKLX59394 | SRR15460883 | CC59 | 59 | SCC*mec* IVa (2B) | t437 | MRSA | 2016 |
| SKLX59398 | SRR15460882 | CC59 | 59 | SCC*mec* IVa (2B) | t437 | MRSA | 2016 |
| SKLX59439 | SRR15460881 | CC59 | 59 | SCC*mec* IVa (2B) | t3517 | MRSA | 2016 |
| SKLX59465 | SRR15460880 | CC59 | 59 | SCC*mec* IVa (2B) | t172 | MRSA | 2016 |
| SKLX59491 | SRR15460879 | CC59 | 59 | SCC*mec* IVa (2B) | t437 | MRSA | 2016 |
| SKLX59513 | SRR15460878 | CC5 | 965 | SCC*mec* IVc (2B) | t062 | MRSA | 2016 |
| SKLX59525 | SRR15460877 | CC45 | 508 | SCC*mec* IVi (2B) | t1608 | MRSA | 2016 |
| SKLX59537 | SRR15460876 | CC8 | 239 | SCC*mec* III (3A) | t037 | MRSA | 2016 |
| SKLX59541 | SRR15460875 | CC8 | 239 | SCC*mec* III (3A) | t037 | MRSA | 2016 |
| SKLX59546 | SRR15460873 | CC59 | 59 | SCC*mec* IVg (2B) | t437 | MRSA | 2016 |
| SKLX59550 | SRR15460872 | CC59 | 59 | SCC*mec* IVa (2B) | t3736 | MRSA | 2016 |
| SKLX59579 | SRR15460871 | CC59 | 59 | SCC*mec* IVa (2B) | t437 | MRSA | 2016 |
| SKLX59589 | SRR15460870 | CC59 | 59 | SCC*mec* IVa (2B) | t437 | MRSA | 2016 |
| SKLX59644 | SRR15460632 | CC59 | 59 | SCC*mec* IVg (2B) | t437 | MRSA | 2016 |
| SKLX59655 | SRR15460631 | CC59 | 59 | SCC*mec* IVg (2B) | t437 | MRSA | 2016 |
| SKLX59887 | SRR15460630 | CC59 | 59 | SCC*mec* IVa (2B) | t437 | MRSA | 2016 |
| SKLX59891 | SRR15460629 | CC88 | 88 | SCC*mec* IVc (2B) | t2310 | MRSA | 2016 |
| SKLX59892 | SRR15460628 | CC59 | 59 | SCC*mec* IVa (2B) | t441 | MRSA | 2016 |
| SKLX59908 | SRR15460627 | CC5 | 5 | SCC*mec* II (2A) | t15309 | MRSA | 2016 |
| SKLX59931 | SRR15460625 | CC59 | 338 | SCC*mec* V (5C2&5) | t437 | CA-MRSA | 2016 |
| SKLX59942 | SRR15460624 | CC59 | 59 | SCC*mec* V (5C2&5) | t437 | MRSA | 2016 |
| SKLX59953 | SRR15460623 | CC59 | 59 | SCC*mec* IVa (2B) | t437 | MRSA | 2016 |
| SKLX59977 | SRR15460622 | CC8 | 239 | SCC*mec* III (3A) | t030 | MRSA | 2016 |
| SKLX59978 | SRR15460621 | CC59 | 338 | SCC*mec* V (5C2&5) | t437 | CA-MRSA | 2016 |
| SKLX59986 | SRR15460620 | CC8 | 239 | SCC*mec* III (3A) | t030 | MRSA | 2016 |
| SKLX60011 | SRR15460619 | CC8 | 239 | SCC*mec* III (3A) | t030 | MRSA | 2016 |
| SKLX60032 | SRR15460618 | CC8 | 239 | SCC*mec* III (3A) | t030 | MRSA | 2016 |
| SKLX60107 | SRR15460617 | CC5 | 965 | SCC*mec* IVc (2B) | t062 | MRSA | 2016 |
| SKLX60120 | SRR15460616 | CC5 | 965 | SCC*mec* IVc (2B) | t062 | MRSA | 2016 |
| SKLX60124 | SRR15460614 | CC5 | 5 | SCC*mec* II (2A) | t311 | HA-MRSA | 2016 |
| SKLX60137 | SRR15460613 | CC5 | 5 | SCC*mec* II (2A) | t311 | MRSA | 2016 |
| SKLX60157 | SRR15460612 | CC59 | 59 | SCC*mec* IVa (2B) | t3523 | MRSA | 2017 |
| SKLX60173 | SRR15460611 | CC5 | 965 | SCC*mec* IVc (2B) | t062 | MRSA | 2017 |
| SKLX60180 | SRR15460610 | CC59 | 59 | SCC*mec* IVa (2B) | t163 | MRSA | 2017 |
| SKLX60185 | SRR15460609 | CC59 | 59 | SCC*mec* IVa (2B) | t437 | MRSA | 2017 |
| SKLX60218 | SRR15460608 | CC5 | 5 | SCC*mec* II (2A) | t2460 | MRSA | 2017 |
| SKLX60246 | SRR15460607 | CC59 | 59 | SCC*mec* V (5C2&5) | t437 | MRSA | 2017 |
| SKLX60260 | SRR15460606 | CC8 | 630 | SCC*mec* V (5C2) | t4549 | MRSA | 2017 |
| SKLX60328 | SRR15460605 | CC59 | 59 | SCC*mec* IVa (2B) | t3527 | MRSA | 2017 |
| SKLX60349 | SRR15460529 | CC59 | 59 | SCC*mec* IVa (2B) | t441 | MRSA | 2017 |
| SKLX60371 | SRR15460528 | CC59 | 338 | SCC*mec* V (5C2&5) | t441 | CA-MRSA | 2017 |
| SKLX60404 | SRR15460527 | CC398 | 398 | SCC*mec* V (5C2) | t034 | MRSA | 2017 |
| SKLX60459 | SRR15460526 | CC59 | 59 | SCC*mec* IVa (2B) | t437 | MRSA | 2017 |
| SRR60471 | SRR15460376 | CC8 | 72 | SCC*mec* IVc (2B) | t324 | MRSA | 2017 |
| SKLX60519 | SRR15460525 | CC5 | 965 | SCC*mec* IVc (2B) | t062 | MRSA | 2017 |
| SKLX60524 | SRR15460524 | CC59 | 59 | SCC*mec* IVa (2B) | t437 | MRSA | 2017 |
| SKLX60527 | SRR15460523 | Unknown | Unknown | SCC*mec* IVa (2B) | t437 | MRSA | 2017 |
| SKLX60530 | SRR15460522 | CC59 | 59 | SCC*mec* IVa (2B) | t437 | MRSA | 2017 |
| SKLX60540 | SRR15460521 | CC59 | 59 | SCC*mec* IVa (2B) | t437 | MRSA | 2017 |
| SKLX60555 | SRR15460520 | CC59 | 59 | SCC*mec* IVa (2B) | t437 | MRSA | 2017 |
| SKLX60562 | SRR15460518 | CC22 | 22 | SCC*mec* V (5C2&5) | t309 | MRSA | 2017 |
| SKLX60713 | SRR15460517 | CC5 | 5529 | SCC*mec* IVg (2B) | t688 | MRSA | 2017 |
| SKLX60733 | SRR15460516 | CC59 | 59 | SCC*mec* IVa (2B) | t437 | MRSA | 2017 |
| SKLX60779 | SRR15460515 | CC59 | 59 | SCC*mec* IVa (2B) | t441 | MRSA | 2017 |
| SKLX60806 | SRR15460514 | CC8 | 239 | SCC*mec* III (3A) | t030 | MRSA | 2017 |
| SKLX60811 | SRR15460513 | CC59 | 59 | SCC*mec* IVa (2B) | t437 | MRSA | 2017 |
| SKLX60813 | SRR15460512 | CC8 | 239 | SCC*mec* III (3A) | t030 | MRSA | 2017 |
| SKLX60822 | SRR15460511 | CC8 | 239 | SCC*mec* III (3A) | t030 | MRSA | 2017 |
| SKLX60835 | SRR15460510 | CC8 | 239 | SCC*mec* III (3A) | t030 | MRSA | 2017 |
| SKLX60840 | SRR15460509 | CC8 | 239 | SCC*mec* III (3A) | t030 | MRSA | 2017 |
| SKLX60915 | SRR15460506 | CC59 | 59 | SCC*mec* IVa (2B) | t163 | MRSA | 2017 |
| SKLX61006 | SRR15460505 | CC8 | 239 | SCC*mec* III (3A) | t030 | MRSA | 2017 |
| SKLX61008 | SRR15460504 | CC8 | 239 | SCC*mec* III (3A) | t030 | MRSA | 2017 |
| SKLX61010 | SRR15460503 | CC59 | 59 | SCC*mec* IVa (2B) | t437 | MRSA | 2017 |
| SKLX61063 | SRR15460390 | CC5 | 5 | SCC*mec* II (2A) | t311 | MRSA | 2017 |
| SKLX61080 | SRR15460389 | Unknown | Unknown | SCC*mec* IVa (2B) | t437 | MRSA | 2017 |
| SKLX61098 | SRR15460388 | CC5 | 5 | SCC*mec* II (2A) | t002 | MRSA | 2017 |
| SKLX61197 | SRR15460387 | CC59 | 59 | SCC*mec* IVa (2B) | t3401 | MRSA | 2017 |
| SKLX61251 | SRR15460386 | CC59 | 59 | SCC*mec* IVa (2B) | t437 | MRSA | 2017 |
| SKLX61303 | SRR15460385 | CC59 | 59 | SCC*mec* V (5C2&5) | t437 | MRSA | 2017 |
| SKLX61304 | SRR15460383 | CC59 | 59 | SCC*mec* IVa (2B) | t437 | MRSA | 2017 |
| SKLX61321 | SRR15460382 | CC88 | 88 | Unkown | t7637 | MRSA | 2017 |
| SKLX61339 | SRR15460381 | CC59 | 59 | SCC*mec* IVa (2B) | t7281 | MRSA | 2017 |
| SKLX61340 | SRR15460380 | CC509 | 509 | SCC*mec* IVa (2B) | t375 | MRSA | 2017 |
| SKLX61370 | SRR15460379 | CC88 | 88 | SCC*mec* IVc (2B) | t2310 | MRSA | 2017 |
| SKLX61393 | SRR15460378 | CC59 | 59 | SCC*mec* V (5C2&5) | t437 | MRSA | 2017 |
| SKLX61416 | SRR15460377 | CC398 | 398 | Unkown | t034 | MRSA | 2017 |
| SKLX61436 | SRR15460375 | Unknown | Unknown | SCC*mec* IVa (2B) | t437 | MRSA | 2017 |
| SKLX61437 | SRR15460826 | Unknown | Unknown | SCC*mec* IVa (2B) | t437 | MRSA | 2017 |
| SKLX61583 | SRR15460825 | CC59 | 59 | SCC*mec* IVa (2B) | t437 | MRSA | 2017 |
| SKLX61654 | SRR15460823 | CC8 | 239 | SCC*mec* III (3A) | t030 | MRSA | 2017 |
| SKLX61759 | SRR15460822 | CC5 | 5 | SCC*mec* II (2A) | t311 | HA-MRSA | 2017 |
| SKLX61826 | SRR15460821 | CC88 | 88 | SCC*mec* IVc (2B) | t2310 | MRSA | 2017 |
| SKLX61885 | SRR15460820 | CC398 | 398 | SCC*mec* V (5C2) | t571 | MRSA | 2017 |
| SKLX61912 | SRR15460819 | CC88 | 88 | SCC*mec* IVc (2B) | t7480 | MRSA | 2017 |
| SKLX61977 | SRR15460818 | Unknown | Unknown | SCC*mec* IVa (2B) | t17857 | MRSA | 2017 |
| SKLX62137 | SRR15460817 | CC8 | 239 | SCC*mec* III (3A) | t030 | MRSA | 2017 |
| SKLX62287 | SRR15460816 | Unknown | Unknown | SCC*mec* II (2A) | t9353 | MRSA | 2017 |
| SKLX62471 | SRR15460815 | CC1 | 9 | SCC*mec* XII (9C2) | t899 | MRSA | 2017 |
| SKLX62563 | SRR15460814 | CC8 | 239 | SCC*mec* III (3A) | t632 | MRSA | 2017 |
| SKLX62574 | SRR15461067 | CC59 | 59 | SCC*mec* IVa (2B) | t437 | MRSA | 2017 |
| SKLX62660 | SRR15461066 | CC59 | 59 | SCC*mec* IVa (2B) | t3523 | MRSA | 2018 |
| SKLX63041 | SRR15461065 | CC88 | 88 | Unkown | t14340 | MRSA | 2018 |
| SKLX63045 | SRR15461064 | CC88 | 88 | Unkown | t14340 | MRSA | 2018 |
| SKLX63071 | SRR15461063 | Unknown | Unknown | SCC*mec* V (5C2&5) | t437 | MRSA | 2018 |
| SKLX63091 | SRR15461062 | CC398 | 398 | SCC*mec* V (5C2) | t034 | MRSA | 2018 |
| SKLX63108 | SRR15461061 | CC8 | 630 | SCC*mec* V (5C2) | t4549 | MRSA | 2018 |
| SKLX63133 | SRR15461060 | CC59 | 59 | SCC*mec* IVa (2B) | t3424 | MRSA | 2018 |
| SKLX63203 | SRR15461059 | Unknown | Unknown | SCC*mec* IVa (2B) | t437 | MRSA | 2018 |
| SKLX63254 | SRR15461058 | Unknown | Unknown | SCC*mec* IVa (2B) | t163 | MRSA | 2018 |
| SKLX63323 | SRR15461056 | CC59 | 59 | SCC*mec* IVa (2B) | t172 | MRSA | 2018 |
| SKLX63326 | SRR15461055 | CC8 | 630 | SCC*mec* V (5C2&5) | t4549 | MRSA | 2018 |
| SKLX63327 | SRR15461054 | CC59 | 59 | SCC*mec* IVa (2B) | t3523 | MRSA | 2018 |
| SKLX63329 | SRR15461053 | CC88 | 88 | Unkown | t14340 | MRSA | 2018 |
| SKLX63331 | SRR15461052 | CC5 | 764 | SCC*mec* II (2A) | t002 | MRSA | 2018 |
| SKLX63333 | SRR15461051 | CC59 | 59 | SCC*mec* IVa (2B) | t3517 | MRSA | 2018 |
| SKLX63348 | SRR15461050 | CC59 | 59 | SCC*mec* IVa (2B) | t437 | MRSA | 2018 |
| SKLX63372 | SRR15461049 | CC59 | 59 | SCC*mec* IVa (2B) | t437 | MRSA | 2018 |
| SKLX63559 | SRR15461048 | CC5 | 5 | SCC*mec* II (2A) | t2460 | MRSA | 2018 |
| SKLX63565 | SRR15461047 | CC5 | 5 | SCC*mec* II (2A) | t2460 | MRSA | 2018 |
| SKLX63580 | SRR15461045 | CC398 | 398 | SCC*mec* V (5C2) | t034 | MRSA | 2018 |
| SKLX63727 | SRR15461044 | CC59 | 59 | SCC*mec* IVa (2B) | t172 | MRSA | 2018 |
| SKLX63734 | SRR15461043 | CC5 | 5 | SCC*mec* II (2A) | t311 | MRSA | 2018 |
| SKLX63744 | SRR15461042 | CC398 | 398 | SCC*mec* V (5C2) | t1928 | MRSA | 2018 |
| SKLX63752 | SRR15461041 | CC1 | 1 | SCC*mec* IVa (2B) | t127 | MRSA | 2018 |
| SKLX63877 | SRR15460869 | CC398 | 398 | SCC*mec* V (5C2) | t034 | MRSA | 2018 |
| SKLX63907 | SRR15460868 | CC59 | 59 | SCC*mec* IVa (2B) | t4669 | MRSA | 2018 |
| SKLX63908 | SRR15460867 | CC59 | 59 | SCC*mec* V (5C2&5) | t437 | MRSA | 2018 |
| SKLX64029 | SRR15460866 | CC5 | 5 | SCC*mec* II (2A) | t311 | MRSA | 2017 |
| SKLX64992 | SRR15460865 | CC8 | 630 | SCC*mec* V (5C2&5) | t4549 | MRSA | 2017 |
| SKLX65073 | SRR15460863 | CC1 | 1 | SCC*mec* IVa (2B) | t127 | MRSA | 2017 |
| SKLX65332 | SRR15460862 | CC59 | 59 | SCC*mec* IVa (2B) | t437 | MRSA | 2017 |
| SKLX67162 | SRR15460861 | CC59 | 59 | SCC*mec* IVa (2B) | t437 | MRSA | 2017 |
| SKLX68096 | SRR15460860 | CC59 | 59 | SCC*mec* IVa (2B) | t172 | MRSA | 2017 |
| SKLX68675 | SRR15460859 | CC5 | 5 | SCC*mec* II (2A) | t311 | MRSA | 2017 |
| SKLX69629 | SRR15460858 | CC5 | 5 | SCC*mec* II (2A) | t002 | HA-MRSA | 2018 |
| SKLX69820 | SRR15460857 | CC5 | 5 | SCC*mec* II (2A) | None | MRSA | 2018 |
| SKLX70159 | SRR15460856 | CC398 | 398 | SCC*mec* V (5C2) | t034 | MRSA | 2018 |
| SKLX70335 | SRR15460855 | CC59 | 59 | SCC*mec* IVa (2B) | t437 | CA-MRSA | 2018 |
| SKLX70355 | SRR15460854 | CC5 | 5 | SCC*mec* II (2A) | None | HA-MRSA | 2018 |
| SKLX70439 | SRR15460852 | CC7 | 7 | SCC*mec* V (5C2&5) | t091 | MRSA | 2018 |
| SKLX70821 | SRR15460851 | CC59 | 59 | SCC*mec* IVa (2B) | t172 | CA-MRSA | 2018 |
| SKLX70993 | SRR15460850 | CC5 | 965 | SCC*mec* IVa (2B) | t062 | MRSA | 2018 |
| SKLX71502 | SRR15460849 | Unknown | Unknown | SCC*mec* II (2A) | t3235 | MRSA | 2018 |
| SKLX72486 | SRR15460848 | CC5 | 5 | SCC*mec* II (2A) | t311 | MRSA | 2018 |
| SKLX72566 | SRR15460847 | CC5 | 5 | SCC*mec* II (2A) | t311 | MRSA | 2018 |
| SKLX74659 | SRR15460846 | CC88 | 88 | SCC*mec* IVc (2B) | t15074 | MRSA | 2018 |
| SKLX75802 | SRR15460845 | CC5 | 5 | SCC*mec* II (2A) | t311 | MRSA | 2018 |
| SKLX75869 | SRR15460844 | CC398 | 398 | SCC*mec* V (5C2) | t011 | MRSA | 2018 |
| SKLX76788 | SRR15460843 | CC1 | 1 | SCC*mec* V (5C2) | t127 | MRSA | 2018 |
| SKLX76890 | SRR15460576 | CC5 | 5 | SCC*mec* II (2A) | t311 | HA-MRSA | 2018 |
| SKLX77120 | SRR15460575 | CC5 | 5 | SCC*mec* II (2A) | t311 | MRSA | 2018 |
| SKLX77782 | SRR15460574 | CC59 | 59 | SCC*mec* V (5C2&5) | t437 | MRSA | 2018 |
| SKLX77838 | SRR15460573 | CC5 | 5 | SCC*mec* II (2A) | t2460 | MRSA | 2018 |
| SKLX78048 | SRR15460572 | CC5 | 5 | SCC*mec* II (2A) | t2460 | HA-MRSA | 2018 |
| SKLX78407 | SRR15460571 | CC5 | 5 | SCC*mec* II (2A) | t2460 | MRSA | 2018 |
| SKLX78421 | SRR15460570 | CC5 | 5 | SCC*mec* II (2A) | t2460 | MRSA | 2018 |
| SKLX78422 | SRR15460569 | CC59 | 59 | SCC*mec* IVa (2B) | t437 | MRSA | 2018 |
| SKLX78437 | SRR15460568 | CC5 | 5 | SCC*mec* II (2A) | t2460 | MRSA | 2018 |
| SKLX78468 | SRR15460567 | CC59 | 59 | SCC*mec* IVc (2B) | t441 | MRSA | 2017 |
| SKLX78502 | SRR15460565 | CC59 | 59 | SCC*mec* IVa (2B) | t437 | MRSA | 2018 |
| SKLX78504 | SRR15460564 | CC59 | 59 | SCC*mec* IVa (2B) | t441 | MRSA | 2018 |
| SKLX78512 | SRR15460563 | CC1 | 9 | SCC*mec* XII (9C2) | t899 | MRSA | 2018 |
| SKLX78523 | SRR15460562 | CC59 | 59 | SCC*mec* IVa (2B) | t1146 | MRSA | 2018 |
| SKLX78557 | SRR15460561 | CC59 | 59 | SCC*mec* IVa (2B) | t437 | MRSA | 2018 |
| SKLX79131 | SRR15460560 | CC59 | 59 | SCC*mec* IVa (2B) | t437 | MRSA | 2018 |
| SKLX79174 | SRR15460559 | CC121 | 121 | SCC*mec* V (5C2&5) | t8660 | MRSA | 2018 |
| SKLX79236 | SRR15460558 | CC59 | 59 | SCC*mec* IVa (2B) | t437 | MRSA | 2017 |
| SKLX79363 | SRR15460557 | CC59 | 59 | SCC*mec* IVa (2B) | t441 | MRSA | 2017 |
| SKLX79370 | SRR15460556 | CC5 | 5 | SCC*mec* II (2A) | t311 | HA-MRSA | 2017 |
| SKLX79371 | SRR15460553 | CC59 | 59 | SCC*mec* V (5C2&5) | t437 | CA-MRSA | 2017 |
| SKLX79376 | SRR15460552 | CC5 | 5 | SCC*mec* II (2A) | t311 | HA-MRSA | 2017 |
| SKLX79391 | SRR15460551 | CC5 | 5 | SCC*mec* II (2A) | t311 | HA-MRSA | 2017 |
| SKLX79455 | SRR15460550 | CC5 | 5 | SCC*mec* II (2A) | t311 | MRSA | 2017 |
| SKLX79473 | SRR15460549 | CC5 | 965 | SCC*mec* IVc (2B) | t062 | MRSA | 2017 |
| SKLX79475 | SRR15460418 | CC5 | 5 | SCC*mec* II (2A) | t311 | MRSA | 2017 |
| SKLX79477 | SRR15460417 | CC59 | 59 | SCC*mec* IVa (2B) | t441 | MRSA | 2017 |
| SKLX79543 | SRR15460416 | CC1 | 188 | SCC*mec* IVa (2B) | t189 | MRSA | 2017 |
| SRR79556 | SRR15460673 | CC8 | 72 | SCC*mec* V (5C2&5) | t148 | MRSA | 2017 |
| SKLX79593 | SRR15460415 | CC5 | 965 | SCC*mec* IVc (2B) | t062 | MRSA | 2017 |
| SKLX79611 | SRR15460414 | CC1 | 1 | SCC*mec* IVg (2B) | t114 | MRSA | 2017 |
| SKLX79620 | SRR15460412 | CC59 | 59 | SCC*mec* IVa (2B) | t172 | MRSA | 2017 |
| SKLX79682 | SRR15460411 | CC59 | 59 | SCC*mec* IVa (2B) | t437 | CA-MRSA | 2018 |
| SKLX79687 | SRR15460410 | CC5 | 5 | SCC*mec* II (2A) | t311 | HA-MRSA | 2018 |
| SKLX79700 | SRR15460409 | CC5 | 965 | SCC*mec* IVa (2B) | t062 | MRSA | 2018 |
| SKLX79708 | SRR15460408 | CC5 | 5 | SCC*mec* II (2A) | t311 | HA-MRSA | 2018 |
| SKLX79710 | SRR15460407 | CC5 | 965 | SCC*mec* IVa (2B) | t062 | MRSA | 2018 |
| SKLX79729 | SRR15460406 | CC5 | 965 | SCC*mec* IVc (2B) | t062 | MRSA | 2018 |
| SKLX79732 | SRR15460405 | CC5 | 965 | SCC*mec* IVc (2B) | t062 | MRSA | 2018 |
| SKLX79735 | SRR15460404 | CC5 | 5 | SCC*mec* II (2A) | t311 | HA-MRSA | 2018 |
| SKLX79749 | SRR15460403 | CC5 | 5 | SCC*mec* II (2A) | t311 | HA-MRSA | 2018 |
| SKLX79752 | SRR15460401 | CC5 | 965 | SCC*mec* IVc (2B) | t062 | MRSA | 2018 |
| SKLX79769 | SRR15460400 | CC5 | 965 | SCC*mec* IVc (2B) | t062 | MRSA | 2018 |
| SKLX79793 | SRR15460399 | CC5 | 965 | SCC*mec* IVc (2B) | t062 | MRSA | 2018 |
| SKLX80731 | SRR15460398 | CC22 | 22 | SCC*mec* V (5C2&5) | t309 | MRSA | 2018 |
| SKLX80737 | SRR15460397 | CC88 | 88 | SCC*mec* V (5C2) | t2526 | MRSA | 2018 |
| SKLX80913 | SRR15460396 | CC59 | 59 | SCC*mec* IVa (2B) | t437 | MRSA | 2018 |
| SKLX81100 | SRR15460395 | CC59 | 59 | SCC*mec* IVa (2B) | t441 | MRSA | 2018 |
| SKLX81101 | SRR15460394 | CC88 | 88 | Unkown | t7637 | MRSA | 2018 |
| SKLX81115 | SRR15460393 | CC59 | 59 | SCC*mec* IVa (2B) | t437 | MRSA | 2018 |
| SKLX81207 | SRR15460392 | CC5 | 5 | SCC*mec* II (2A) | t311 | MRSA | 2018 |
| SKLX81208 | SRR15460785 | CC5 | 5 | SCC*mec* II (2A) | t1215 | HA-MRSA | 2018 |
| SKLX81408 | SRR15460784 | CC5 | 5 | SCC*mec* II (2A) | t1215 | HA-MRSA | 2018 |
| SKLX81560 | SRR15460783 | CC8 | 630 | SCC*mec* V (5C2) | t4549 | MRSA | 2018 |
| SKLX81650 | SRR15460782 | CC5 | 5 | SCC*mec* II (2A) | t1215 | MRSA | 2018 |
| SKLX81729 | SRR15460781 | CC59 | 59 | SCC*mec* IVa (2B) | t437 | MRSA | 2018 |
| SKLX81816 | SRR15460780 | CC59 | 59 | SCC*mec* V (5C2&5) | t437 | MRSA | 2018 |
| SKLX82063 | SRR15460779 | CC59 | 59 | SCC*mec* IVa (2B) | t437 | MRSA | 2018 |
| SKLX82774 | SRR15460778 | CC45 | 508 | SCC*mec* IVi (2B) | t015 | MRSA | 2018 |
| SKLX82790 | SRR15460777 | CC59 | 338 | SCC*mec* V (5C2&5) | t437 | CA-MRSA | 2018 |
| SKLX82812 | SRR15460776 | CC1 | 9 | SCC*mec* XII (9C2) | t899 | MRSA | 2018 |
| SKLX82849 | SRR15460774 | CC59 | 59 | SCC*mec* IVa (2B) | t437 | MRSA | 2018 |
| SKLX82898 | SRR15460773 | CC59 | 59 | SCC*mec* IVa (2B) | t437 | MRSA | 2018 |
| SKLX82906 | SRR15460772 | CC398 | 398 | SCC*mec* V (5C2) | t034 | MRSA | 2018 |
| SKLX82910 | SRR15460771 | CC59 | 59 | SCC*mec* IVa (2B) | t437 | MRSA | 2018 |
| SKLX82911 | SRR15460770 | CC59 | 59 | SCC*mec* IVa (2B) | t437 | MRSA | 2018 |
| SKLX82998 | SRR15460769 | CC59 | 59 | SCC*mec* IVa (2B) | t437 | MRSA | 2018 |
| SKLX83000 | SRR15460768 | Unknown | Unknown | SCC*mec* IVa (2B) | t172 | MRSA | 2018 |
| SKLX83024 | SRR15460767 | CC59 | 4513 | SCC*mec* IVa (2B) | t437 | MRSA | 2018 |
| SKLX83059 | SRR15460766 | CC22 | 22 | SCC*mec* V (5C2&5) | t309 | MRSA | 2018 |
| SKLX83072 | SRR15460765 | CC59 | 59 | SCC*mec* IVg (2B) | t437 | MRSA | 2018 |
| SKLX83133 | SRR15460763 | CC398 | 398 | SCC*mec* V (5C2) | t2876 | MRSA | 2018 |
| SKLX83183 | SRR15460762 | CC59 | 59 | SCC*mec* IVa (2B) | t437 | MRSA | 2018 |
| SKLX83220 | SRR15460761 | CC5 | 5 | SCC*mec* II (2A) | t311 | MRSA | 2018 |
| SKLX83686 | SRR15460760 | CC8 | 239 | SCC*mec* III (3A) | t030 | MRSA | 2018 |
| SKLX83688 | SRR15460759 | CC59 | 59 | SCC*mec* IVa (2B) | t441 | MRSA | 2018 |
| SKLX83689 | SRR15460758 | CC8 | 239 | SCC*mec* III (3A) | t030 | MRSA | 2018 |
| SKLX83693 | SRR15460729 | CC5 | 5 | SCC*mec* II (2A) | t002 | MRSA | 2018 |
| SKLX83694 | SRR15460728 | CC5 | 764 | SCC*mec* II (2A) | t002 | MRSA | 2018 |
| SKLX83700 | SRR15460727 | CC5 | 5 | SCC*mec* II (2A) | t2460 | MRSA | 2018 |
| SKLX83809 | SRR15460726 | CC59 | 59 | SCC*mec* IVa (2B) | t437 | MRSA | 2018 |
| SKLX83810 | SRR15460724 | CC5 | 5 | SCC*mec* IVg (2B) | t688 | MRSA | 2018 |
| SKLX83812 | SRR15460723 | Unknown | Unknown | SCC*mec* IVa (2B) | t172 | MRSA | 2018 |
| SKLX83818 | SRR15460722 | CC59 | 59 | SCC*mec* IVa (2B) | t437 | MRSA | 2018 |
| SKLX83877 | SRR15460721 | CC59 | 59 | SCC*mec* IVa (2B) | t172 | MRSA | 2018 |
| SRR83971 | SRR15460672 | CC8 | 72 | SCC*mec* IVc (2B) | t324 | MRSA | 2018 |
| SRR83977 | SRR15460671 | CC8 | 72 | SCC*mec* IVc (2B) | t324 | MRSA | 2018 |
| SKLX84007 | SRR15460720 | CC59 | 59 | SCC*mec* IVa (2B) | t437 | MRSA | 2018 |
| SKLX84046 | SRR15460719 | CC398 | 398 | SCC*mec* V (5C2) | t034 | MRSA | 2018 |
| SKLX84669 | SRR15460718 | CC5 | 5 | SCC*mec* II (2A) | t311 | HA-MRSA | 2018 |
| SKLX84856 | SRR15460717 | CC59 | 59 | SCC*mec* IVa (2B) | t172 | MRSA | 2018 |
| SKLX84976 | SRR15460716 | CC5 | 5 | SCC*mec* II (2A) | t311 | MRSA | 2018 |
| SKLX85152 | SRR15460715 | CC59 | 59 | SCC*mec* IVa (2B) | t3517 | MRSA | 2018 |
| SKLX85755 | SRR15460713 | CC59 | 3260 | SCC*mec* V (5C2&5) | t437 | MRSA | 2018 |
| SKLX85887 | SRR15460712 | CC59 | 59 | SCC*mec* IVa (2B) | t437 | MRSA | 2018 |
| SKLX87136 | SRR15460711 | CC59 | 59 | SCC*mec* V (5C2&5) | t437 | MRSA | 2018 |
| SKLX87194 | SRR15460710 | Unknown | Unknown | SCC*mec* V (5C2&5) | t437 | MRSA | 2018 |
| SKLX87214 | SRR15460709 | CC59 | 3031 | SCC*mec* IVa (2B) | t14062 | MRSA | 2018 |
| SKLX87248 | SRR15460708 | CC5 | 5 | SCC*mec* II (2A) | t311 | MRSA | 2018 |
| SKLX87346 | SRR15460707 | CC1 | 1 | SCC*mec* IVa (2B) | t127 | MRSA | 2018 |
| SKLX87673 | SRR15460706 | CC398 | 398 | SCC*mec* V (5C2) | t1456 | MRSA | 2019 |
| SKLX87764 | SRR15460705 | CC59 | 59 | SCC*mec* IVa (2B) | t441 | MRSA | 2019 |
| SKLX87779 | SRR15460704 | CC59 | 59 | SCC*mec* IVa (2B) | t437 | MRSA | 2019 |
| SKLX87780 | SRR15460702 | CC59 | 59 | SCC*mec* IVa (2B) | t437 | MRSA | 2019 |
| SKLX87799 | SRR15461037 | CC59 | 59 | SCC*mec* IVa (2B) | t163 | MRSA | 2019 |
| SKLX87805 | SRR15461036 | CC59 | 59 | SCC*mec* IVa (2B) | t437 | MRSA | 2019 |
| SKLX87900 | SRR15461035 | Unknown | Unknown | SCC*mec* IVa (2B) | t437 | MRSA | 2019 |
| SKLX88115 | SRR15461034 | CC5 | 5 | SCC*mec* II (2A) | t002 | HA-MRSA | 2019 |
| SKLX88401 | SRR15461033 | CC398 | 398 | SCC*mec* V (5C2) | t034 | MRSA | 2019 |
| SKLX88605 | SRR15461032 | CC398 | 398 | SCC*mec* V (5C2) | t034 | MRSA | 2019 |
| SKLX88663 | SRR15461031 | CC59 | 59 | SCC*mec* V (5C2&5) | t437 | MRSA | 2019 |
| SKLX88750 | SRR15461030 | CC5 | 5 | SCC*mec* II (2A) | t311 | MRSA | 2019 |
| SKLX88790 | SRR15461029 | CC5 | 965 | SCC*mec* IVc (2B) | t062 | MRSA | 2019 |
| SKLX88894 | SRR15461027 | CC8 | 630 | SCC*mec* V (5C2&5) | t4549 | MRSA | 2019 |
| SKLX88955 | SRR15461026 | CC88 | 88 | SCC*mec* IVc (2B) | t2310 | MRSA | 2019 |
| SKLX89005 | SRR15461025 | CC5 | 965 | SCC*mec* IVc (2B) | t062 | MRSA | 2019 |
| SKLX89268 | SRR15461024 | CC8 | 1821 | SCC*mec* V (5C2&5) | t4549 | MRSA | 2019 |
| SKLX89362 | SRR15461023 | CC121 | 946 | SCC*mec* IV (2B&5) | t758 | MRSA | 2019 |
| SKLX89369 | SRR15461022 | Unknown | Unknown | SCC*mec* V (5C2&5) | t3592 | MRSA | 2019 |
| SKLX89377 | SRR15461021 | Unknown | Unknown | SCC*mec* V (5C2) | t034 | MRSA | 2019 |
| SKLX89397 | SRR15461020 | CC59 | 3193 | SCC*mec* IVa (2B) | t172 | MRSA | 2019 |
| SKLX89446 | SRR15461019 | Unknown | Unknown | SCC*mec* V (5C2) | t034 | MRSA | 2019 |
| SKLX89512 | SRR15461018 | CC398 | 398 | SCC*mec* V (5C2) | t034 | MRSA | 2019 |
| SKLX89726 | SRR15461015 | CC45 | 45 | SCC*mec* IVa (2B) | t073 | MRSA | 2019 |
| SKLX89771 | SRR15461014 | CC1 | 1 | SCC*mec* IVa (2B) | t127 | MRSA | 2019 |
| SKLX90707 | SRR15461013 | CC59 | 59 | SCC*mec* IVa (2B) | t437 | MRSA | 2019 |
| SKLX91455 | SRR15461012 | CC88 | 88 | SCC*mec* IVc (2B) | t2310 | MRSA | 2019 |
| SKLX91456 | SRR15461011 | CC88 | 88 | Unkown | None | MRSA | 2019 |
| SKLX91461 | SRR15461010 | CC88 | 88 | Unkown | None | MRSA | 2019 |
| SKLX91914 | SRR15460981 | CC5 | 5 | SCC*mec* II (2A) | t311 | HA-MRSA | 2019 |
| SKLX92185 | SRR15460980 | CC5 | 5 | SCC*mec* II (2A) | t311 | HA-MRSA | 2019 |
| SKLX94087 | SRR15460979 | CC5 | 764 | SCC*mec* II (2A) | t002 | MRSA | 2019 |
| SKLX94245 | SRR15460978 | CC59 | 59 | SCC*mec* IVa (2B) | t163 | MRSA | 2019 |
| SKLX94275 | SRR15460976 | CC1 | 188 | SCC*mec* IVa (2B) | t189 | MRSA | 2019 |
| SKLX94276 | SRR15460975 | CC59 | 338 | SCC*mec* V (5C2&5) | t437 | CA-MRSA | 2019 |
| SKLX94289 | SRR15460974 | CC59 | 338 | SCC*mec* V (5C2&5) | t437 | CA-MRSA | 2019 |
| SKLX94291 | SRR15460973 | CC1 | 188 | SCC*mec* IVa (2B) | t189 | MRSA | 2019 |
| SKLX94343 | SRR15460972 | CC59 | 59 | SCC*mec* V (5C2&5) | t437 | MRSA | 2019 |
| SKLX94352 | SRR15460971 | CC59 | 59 | SCC*mec* V (5C2&5) | t437 | MRSA | 2019 |
| SKLX94422 | SRR15460970 | CC5 | 965 | SCC*mec* IVa (2B) | t1399 | MRSA | 2019 |
| SKLX94652 | SRR15460969 | CC398 | 398 | SCC*mec* V (5C2) | t034 | MRSA | 2019 |
| SKLX94661 | SRR15460968 | CC398 | 398 | SCC*mec* V (5C2) | t034 | MRSA | 2019 |
| SKLX94709 | SRR15460967 | CC59 | 59 | SCC*mec* IVa (2B) | t437 | MRSA | 2019 |
| SRR94718 | SRR15460670 | CC8 | 72 | SCC*mec* IVc (2B) | t2431 | MRSA | 2019 |
| SKLX95282 | SRR15460965 | CC398 | 398 | SCC*mec* V (5C2) | t034 | MRSA | 2019 |
| SKLX95326 | SRR15460964 | CC5 | 5 | SCC*mec* II (2A) | t9353 | MRSA | 2019 |
| SKLX95565 | SRR15460963 | CC59 | 59 | SCC*mec* IVa (2B) | t437 | MRSA | 2019 |
| SKLX97039 | SRR15460962 | CC5 | 5 | SCC*mec* II (2A) | t311 | HA-MRSA | 2019 |
| SKLX97560 | SRR15460961 | CC59 | 59 | SCC*mec* IVa (2B) | t2755 | MRSA | 2019 |
| SKLX97564 | SRR15460960 | CC88 | 88 | Unkown | None | MRSA | 2019 |
| SKLX97572 | SRR15460959 | CC59 | 59 | SCC*mec* IVg (2B) | t437 | MRSA | 2019 |
| SKLX98172 | SRR15460958 | CC88 | 88 | SCC*mec* IVc (2B) | t2310 | MRSA | 2019 |
| SKLX98176 | SRR15460957 | Unknown | Unknown | SCC*mec* IVa (2B) | t437 | MRSA | 2019 |
| SKLX98330 | SRR15460956 | CC5 | 5 | SCC*mec* II (2A) | t002 | HA-MRSA | 2019 |
| SKLX99020 | SRR15460954 | CC398 | 1232 | SCC*mec* V (5C2&5) | t034 | MRSA | 2019 |
| SKLX99406 | SRR15460841 | CC1 | 1 | SCC*mec* IVa (2B) | t127 | MRSA | 2019 |
| SKLX99408 | SRR15460840 | CC8 | 630 | SCC*mec* V (5C2) | t4549 | MRSA | 2019 |
| SKLX99456 | SRR15460839 | CC59 | 59 | SCC*mec* V (5C2&5) | t437 | MRSA | 2019 |
| SKLX99479 | SRR15460838 | CC8 | 239 | SCC*mec* III (3A) | t030 | MRSA | 2019 |
| SKLX99480 | SRR15460837 | CC8 | 239 | SCC*mec* III (3A) | t030 | MRSA | 2019 |
